# Observation of giant acoustoelectric currents in graphene on lead magnesium niobate–lead titanate

**DOI:** 10.1038/s41467-026-74813-3

**Published:** 2026-07-10

**Authors:** Hamed Atashbar, Hakhamanesh Mansoorzare, Tara Jabegu, Sidong Lei, Mary E. Galanko Klemash, Sarah S. Bedair, Reza Abdolvand

**Affiliations:** 1 University of Central Florida, Orlando, FL USA; 2 U.S. Army Combat Capabilities Development Command - Army Research Laboratory, Adelphi, MD USA

**Keywords:** Devices for energy harvesting, Electrical and electronic engineering, Acoustics, Two-dimensional materials, Electronic properties and devices

## Abstract

Acoustoelectric (AE) devices enable coupling between acoustic waves and mobile charge carriers, offering a route toward RF-to-DC conversion, RF power sensing, energy harvesting, and responsive sensing applications. The efficiency of this coupling depends on both the electromechanical properties of the piezoelectric substrate and the transport properties of the conductive film interacting with the surface acoustic wave (SAW). Here an AE SAW delay line platform is presented for generating large AE currents using graphene on lead magnesium niobate–lead titanate (PMN–PT). Leveraging graphene’s high carrier mobility and the strong electromechanical coupling of (111)-cut PMN–PT, this platform achieves giant AE current generation. Additionally, AE responsivity (${{\mathscr{R}}}_{{\rm{AE}}}$) is introduced here as a figure of merit for comparing AE platforms. The platform comprises interdigitated transducers (IDTs) deposited and patterned on the bulk PMN–PT surface for launching SAW and a monolayer graphene film positioned within the SAW propagation region. Experimental measurements confirm generation of AE current and voltage within the SAW passband, with the AE current reaching up to 90.39 µA at an RF input power of 20 dBm, corresponding to an ${{\mathscr{R}}}_{{\rm{AE}}}$ of 7.66 µA/(W·MHz). The reported AE current and AE responsivity outperform most of the previously reported platforms, validating the effectiveness of the proposed approach.

## Abstract (web summary)

Acoustoelectric devices can convert radio-frequency-driven acoustic signals into electronic responses and are useful for energy harvesting and various sensing applications. Here, the authors report the integration of graphene with a lead magnesium niobate–lead titanate surface acoustic wave platform, showing enhanced acoustoelectric currents and responsivity.

## Introduction

The acoustoelectric (AE) effect offers a promising mechanism for energy coupling between acoustic and electronic domains, leading to the development of charge transport devices, highly responsive sensors, and next-generation quantum platforms^1^. In particular, RF-to-DC conversion through direct current (DC) generation from RF-driven acoustic waves enables AE devices for energy harvesting and RF power sensing^2,3^. For example, by directly transferring energy from acoustic waves to mobile charge carriers and generating a DC signal, AE sensors can detect subtle changes in material properties, such as carrier mobility or conductivity, which are often modulated by environmental interactions^4,5^. This makes them attractive for a wide range of on-chip and highly responsive sensing applications, including gas, chemical, and humidity detection^6–8^. For instance, the ability to monitor human respiration with AE sensors^9^ hints at applications in respiratory health (e.g., managing asthma or sleep apnea via continuous breath analysis). On the other hand, by pumping energy from charge carriers to acoustic waves, active AE devices can be implemented and used for developing traveling wave amplifiers^10,11^, frequency mixers^12,13^, and non-reciprocal components^14–17^.

In a piezoelectric medium, an acoustic wave (essentially a traveling train of phonons) naturally generates an accompanying electric field due to the piezoelectric coupling. When a resistive layer such as a graphene film is placed on the surface, said traveling electric field interacts with charge carriers in the film. As a result, momentum is transferred from the wave to the electrons, leading to a drag force that causes a net flow of current. This phenomenon is referred to as the AE current, and its magnitude is largely governed by the piezoelectric properties as well as the mobility and conductivity of the film^18,19^. More specifically, the electromechanical coupling directly determines the efficiency of the AE effect and has to be maximized.

**Fig. 1:**
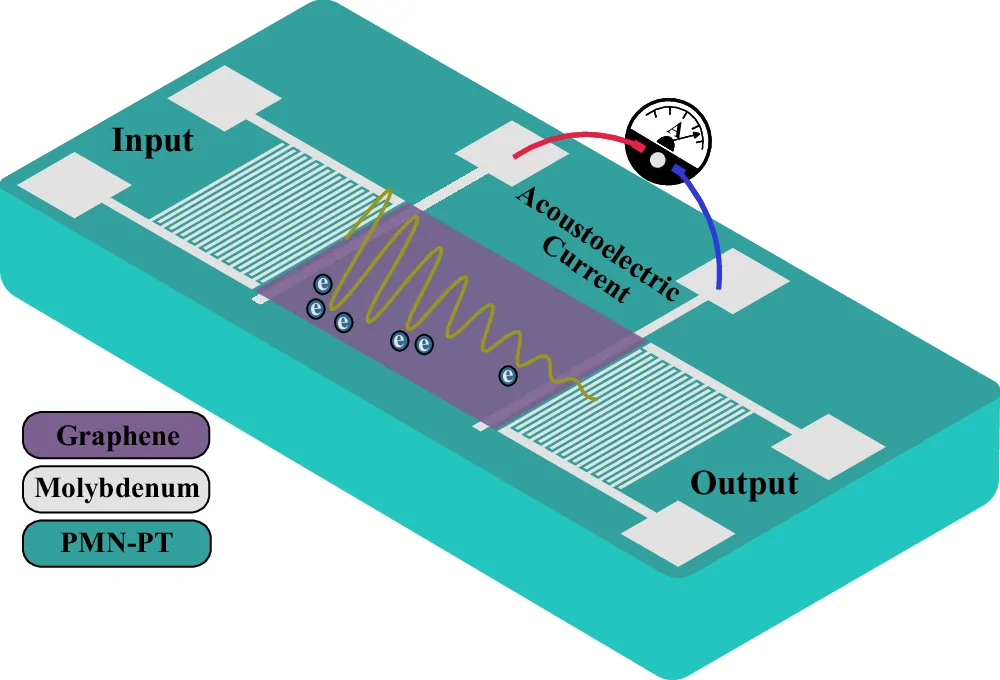
Acoustoelectric surface acoustic wave platform. Schematic of the acoustoelectric (AE) surface acoustic wave (SAW) delay line platform based on graphene integrated on lead magnesium niobate–lead titanate (PMN–PT). Two opposing interdigitated transducers (IDTs) are patterned on the PMN–PT surface to launch and receive SAWs. The purple region represents the graphene film transferred between the IDTs and defines the AE coupling region. The yellow sinusoidal line represents the propagating SAW and its accompanying electric field, which interacts with mobile carriers in graphene. The circles labeled “e” represent electrons in graphene that are dragged by the traveling acoustic wave, producing an AE current measured through the external circuit. Molybdenum electrodes are used for the IDTs and contact pads.

Among conventional piezoelectric materials, lead magnesium niobate–lead titanate (PMN–PT) offers one of the strongest piezoelectric strain constants (${{\rm{d}}}_{22}$), which is relevant to surface acoustic wave (SAW) excitation and subsequently results in a higher electric field intensity accompanying the SAW^20^. Hence, in this work, a SAW delay line platform is investigated based on a bulk PMN–PT substrate and featuring a monolayer graphene film, which is patterned in the SAW delay region, as schematically shown in Fig. 1. Graphene is chosen due to its high carrier mobility and ease of transfer onto the PMN–PT substrate with low thermal budget. The latter is crucial since PMN–PT is a relaxor ferroelectric material which can be depoled at temperatures as low as 50 °C^21^, losing its ferroelectric properties. Among various crystalline cuts of PMN–PT, the (111)-cut is chosen as it has been reported to have the lowest electrical and acoustic loss while providing a high electromechanical coupling^22^.

By combining the high carrier mobility of graphene with the strong piezoelectric coupling of (111)-cut PMN–PT, this platform could enable larger AE currents for a given acoustic power as well as enhanced sensitivity to variations in graphene’s transport properties, such as those induced by gas adsorption or environmental effects. Hence, the main goal of this work is to experimentally quantify the AE current and voltage generated in this platform and verify its efficiency. The measurement results show AE currents as high as 61.17 µA at an RF input power of 20 dBm, which can be further boosted to 90.39 µA by electrostatically tuning the graphene sheet conductivity via a gate voltage. This corresponds to an AE responsivity $({{\mathscr{R}}}_{{\rm{AE}}})$ of 7.66 µA/(W·﻿MHz), introduced here as a figure of merit for meaningful comparison among AE platforms, and defined as AE current (${I}_{{\rm{AE}}}$) normalized to RF input power (${P}_{{\rm{in}}}$) and operating frequency (f), ${{\mathscr{R}}}_{{\rm{AE}}}={I}_{{\rm{AE}}}/({P}_{{\rm{in}}}\cdot f)$. The units of ${{{\mathscr{R}}}}_{{{\rm{AE}}}}$ are A/(W·Hz), equivalently C/W, giving a physically intuitive interpretation as the charge generated per unit input power. These results represent some of the highest AE currents and AE responsivity reported to date, establishing a promising approach for AE-based charge transport and sensing applications.

## Results

### Platform architecture

**Fig. 2:**
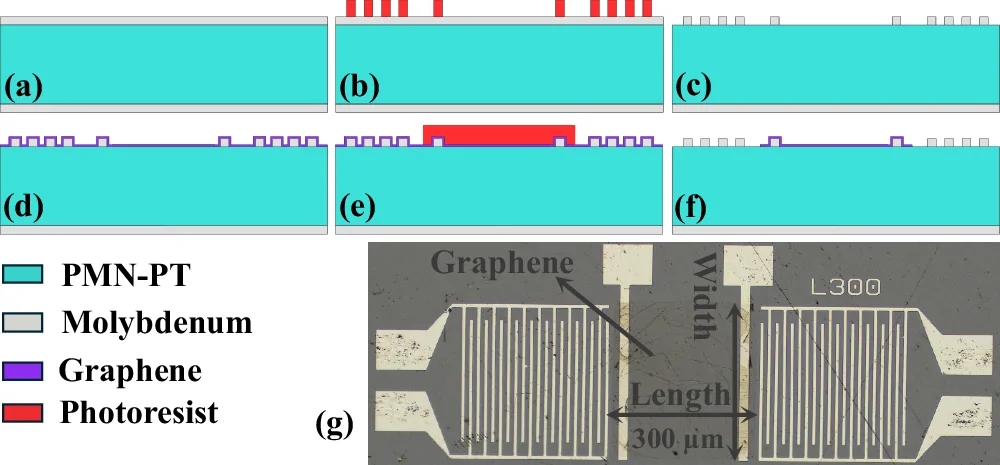
Fabrication process for the graphene-on-PMN–PT device. Simplified fabrication flow for the graphene-on-(111)-cut PMN–PT surface acoustic wave device, together with an optical micrograph of a fabricated device. **a** A molybdenum layer is deposited on the PMN–PT substrate. **b** Photoresist is patterned to define the interdigitated transducers and contact pads. **c** The molybdenum layer is etched to form the surface acoustic wave electrodes. **d** A monolayer graphene film is transferred onto the device surface. **e** A second lithography step defines the graphene coupling region between the two opposing transducers. **f** Oxygen plasma etching removes unwanted graphene, leaving the patterned graphene film between the transducers and connected to the DC readout pads. **g** Optical micrograph of the completed device, showing the two opposing transducers, the graphene coupling region, and the device dimensions used for acoustoelectric measurements.

The SAW delay line comprises two opposing interdigitated transducers (IDTs) separated by an AE coupling region, where a monolayer graphene film interacts with the SAW. Each IDT has 10 finger pairs with a 50% metallization ratio, patterned from a 100-nm-thick molybdenum (Mo) electrode layer, and a pitch of 10 or 15 μm, with corresponding aperture widths of 200 and 300 μm. A monolayer of graphene is transferred onto the coupling region, having a length of 300 μm, with its ends positioned on DC pads for readout. The devices are fabricated on a 500 μm-thick (111)-cut PMN–PT substrate (~30–31% PT content). Fabrication details are provided in the “Methods,” and a representative device micrograph is shown in Fig. 2g.

### Acoustoelectric concept and modeling

For an RF input power (${P}_{{{\rm{in}}}}$) applied to IDTs, the intensity of the launched SAW (I) is proportional to the electromechanical coupling (${K}^{2}$) and defined as acoustic power per unit aperture width (W):

1 $$I=\eta \frac{{K}^{2}{P}_{{{\rm{in}}}}}{W}$$

where η accounts for various loss mechanisms (e.g., impedance mismatch, bidirectional IDT emission). As the SAW propagates beneath the resistive film, momentum is transferred from the SAW to the electrons, leading to SAW attenuation and the generation of an AE current, which has been modeled in prior studies^23,24^. Consequently, SAW intensity decays as of $I={I}_{0}{e}^{-\varGamma x}$ along its propagation path, where ${I}_{0}$ is the SAW intensity on the free piezoelectric surface. This process is described by the classical relaxation model, where the AE attenuation per unit length Γ is given by:

2 $$\varGamma=\frac{\pi {K}^{2}}{\lambda }.\frac{\frac{{\sigma }_{{{\square }}}}{{\sigma }_{{{\rm{m}}}}}}{1+{\left(\frac{{\sigma }_{{{\square }}}}{{\sigma }_{{{\rm{m}}}}}\right)}^{2}}$$

with λ as the SAW wavelength, ${\sigma }_{\square }$ as the sheet conductivity of the resistive film, and ${\sigma }_{{{\rm{m}}}}={v}_{{{\rm{SAW}}}}\,\left(\sqrt{{\varepsilon }_{{{\rm{xx}}}}^{T}\,{\varepsilon }_{{{\rm{zz}}}}^{T}}+1\right){\varepsilon }_{0}$ as a characteristic conductivity determined by SAW velocity and the surrounding dielectric environment. Here, ${\varepsilon }_{{{\rm{xx}}}}^{T}$ and ${\varepsilon }_{{{\rm{zz}}}}^{T}$ denote the dielectric constants of the piezoelectric at constant stress conditions. Maximum AE interaction occurs when the two conductivities match (i.e., ${\sigma }_{{{\square }}}={\sigma }_{{{\rm{m}}}}$). The short-circuit AE current density and open-circuit AE voltage are then:

3 $${j}_{{{\rm{AE}}}}=\mu \frac{I}{{v}_{{{\rm{SAW}}}}}\varGamma,{V}_{{{\rm{AE}}}}={\int }_{0}^{L}\frac{I(x)\varGamma }{{n}_{{{\rm{s}}}}q{v}_{{{\rm{SAW}}}}}{dx}=\frac{{I}_{0}\left(1-{e}^{-\varGamma L}\right)}{{n}_{{{\rm{s}}}}q{v}_{{{\rm{SAW}}}}}$$

where μ is the carrier mobility and the total short-circuit AE current is obtained by integrating over W. In the open-circuit case, the AE voltage builds up until the induced electric field balances the SAW-induced force, where ${n}_{{{\rm{s}}}}$ is the sheet carrier density, q is the elementary charge magnitude and L is the effective interaction length of the SAW and the resistive film.

The AE attenuation can be further enhanced by modulating the properties of the resistive film, providing a tunable parameter for optimizing AE current and voltage. Specifically, when the sheet conductivity of the film exceeds the characteristic conductivity, as is typical for graphene due to its inherently high carrier density and mobility, the AE attenuation is far from optimal (Eq. 2). Graphene exhibits a zero bandgap^25^, with its conduction and valence bands meeting at the Dirac point. Previous works have modeled how a back-gate voltage (${V}_{{bg}}$) can reduce sheet conductivity by electrostatically tuning the carrier density toward the Dirac point^26,27^. Charge carrier density in the gated graphene channel and graphene sheet conductivity are described by:

4 $$n=\frac{{C}_{{{\rm{g}}}}}{q}|{V}_{{{\rm{bg}}}}-{V}_{{{\rm{Dirac}}}}|,{\sigma }_{{{\square }}}=q\mu \sqrt{{n}^{2}+{n}_{0}^{2}},$$

where ${V}_{{{\rm{Dirac}}}}$ is the Dirac point voltage, ${C}_{{{\rm{g}}}}=\frac{{\varepsilon }_{0}{\varepsilon }_{{{\rm{zz}}}}^{T}}{t}$ is the gate capacitance per unit area defined by piezoelectric dielectric constant (${\varepsilon }_{{{\rm{zz}}}}^{T}$) and its thickness (t), and ${n}_{0}$ is the residual carrier density due to charge puddles. Therefore, the sheet conductivity $({\sigma }_{\square })$ versus ${V}_{{{\rm{bg}}}}$ yields a characteristic V-shaped curve, with minimum conductivity at the Dirac point due to minimal charge carrier density. Increasing $|{V}_{{{\rm{bg}}}}-{V}_{{{\rm{Dirac}}}}|$ increases carrier density and conductivity^28^. For ${V}_{{{\rm{bg}}}} > {V}_{{{\rm{Dirac}}}}$, electrons are dominant charge carriers (n-type conduction), while for ${V}_{{{\rm{bg}}}} < {V}_{{{\rm{Dirac}}}}$, holes dominate (p-type conduction). The analytical model predicts that tuning ${\sigma }_{\square }$ toward ${\sigma }_{{{\rm{m}}}}$ by applying ${V}_{{{\rm{bg}}}}$ toward the Dirac point increases Γ and thereby, enhances the AE current and voltage, consistent with experimental observations.

### Graphene characterization

The graphene films are characterized at room temperature and under ambient conditions. The graphene film resistance was first measured via DC terminals, and transferred films exhibited resistance values in the range of 1.3–2.3 kΩ, affected by the non-uniform structure of transferred graphene films. Next, using a Van der Pauw Hall system (MMR Technologies), a room-temperature Hall mobility of $\mu=400.4\,{{{\rm{cm}}}}^{2}/({{\rm{V}}}\cdot {{\rm{s}}})$ was measured for the graphene film; the measurement setup and results are provided in the Supplementary Fig. 3. Next, the effect of applying the back-gate voltage to electrostatically tune the graphene conductivity was studied. After each gate-voltage step, the graphene resistance and the corresponding AE response exhibited a slow relaxation toward steady state; therefore, each bias point was allowed to stabilize before recording the data, typically for about 30 min. This relaxation is likely associated with slow charge trapping/detrapping and interface charge redistribution at the graphene/PMN–PT interface, which can gradually modify the graphene sheet conductivity (Supplementary Note 1)^29^. A bias range of 0–80 V was applied, and the graphene films remained in the p-type regime (i.e., region before charge-neutrality point). This was verified by (1) the measured transfer characteristic showing a monotonic increase in resistance with increasing ${V}_{{{\rm{bg}}}}$ (Supplementary Fig. 1) and (2) observation of no polarity reversal (sign change) of ${I}_{{{\rm{AE}}}}$ as further discussed under the AE response characterizations. Accordingly, the analysis is restricted to a regime sufficiently far from the charge-neutrality point, where the carrier mobility is expected to vary only weakly with ${V}_{{{\rm{bg}}}}$, and is treated as approximately constant^30^.

### SAW characterization and acoustoelectric response

**Fig. 3:**
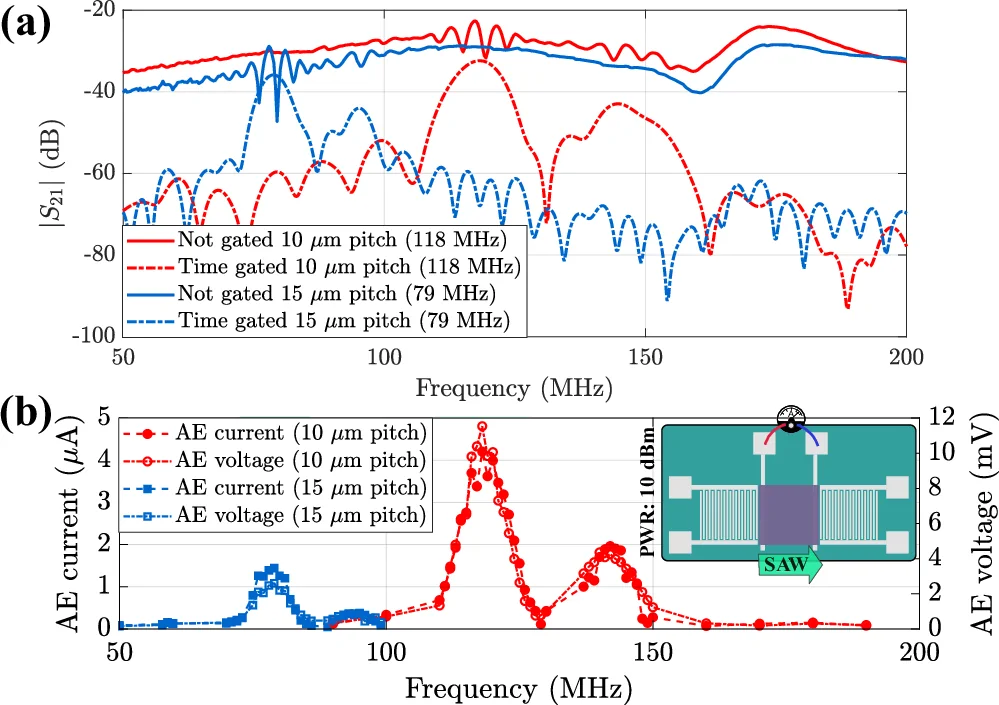
Frequency response of acoustoelectric surface acoustic wave devices. **a** Measured transmission response, |S_21_|, of the SAW delay lines with and without time-gating for devices with 10 μm and 15 μm IDT pitches. Time-gating suppresses electromagnetic feedthrough and isolates the acoustic transmission response, making the SAW passbands more clearly visible near 118 MHz for the 10 μm pitch device and 79 MHz for the 15 μm pitch device. **b** Measured AE current and voltage versus frequency for 10 μm and 15 μm pitch devices at ${P}_{{{\rm{in}}}}=10$ ${{\rm{dBm}}}$, where ${P}_{{{\rm{in}}}}$ is the input power applied to the input IDT. The AE current and voltage peak within the SAW passbands. The inset shows the device configuration used for the measurement, including the input IDT, output IDT, graphene coupling region, SAW propagation direction, and AE readout circuit.

The SAW delay lines are first characterized at room temperature and atmospheric pressure, with the transmission (S_21_) responses shown in Fig. 3a. To better visualize the acoustic passband, measurements were performed with and without time-gating, which applies a time window to isolate the SAW signal from the electromagnetic (EM) feedthrough, yielding a more clear SAW transmission response.

Upon RF excitation, a short-circuit configuration at the graphene DC terminals generates AE current, whereas an open-circuit configuration produces AE voltage across the film. Since the IDTs have a well-defined passband, the SAW intensity and subsequently the AE signal are expected to peak within the passband. This is confirmed by the measurements shown in Fig. 3b, where a 10 dBm RF excitation is swept across the frequency, and strong AE current and voltage peaks are measured aligned with the respective SAW passband frequencies of around 118 MHz (10 μm pitch) and 79 MHz (15 μm pitch). After correcting for the higher transmission loss of the 15 μm pitch device (which results in 2.23× lower SAW intensity), its lower AE current (1.40 vs 4.58 μA) is consistent with Eq. (2), which predicts a reduced Γ, proportional to the λ ratios (λ being twice the pitch, i.e., 15/10 = 1.5×).

**Fig. 4:**
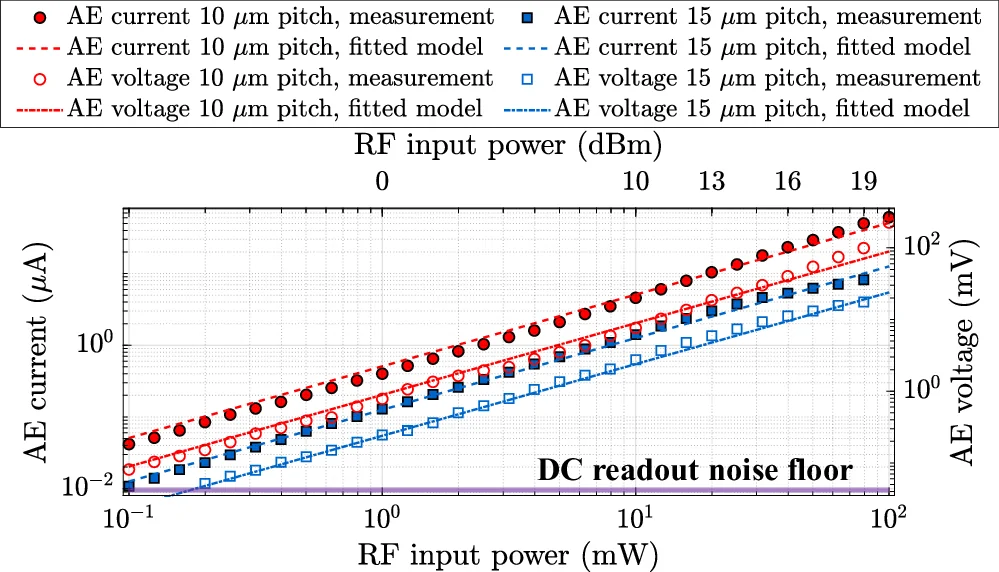
Acoustoelectric current and voltage versus radio frequency input power. Measured AE current and voltage as a function of radio frequency (RF) input power for devices with 10 μm and 15 μm IDT pitches. The bottom axis shows RF input power in milliwatts, while the top axis shows the corresponding power in dBm. Red and blue symbols correspond to the 10 μm and 15 μm pitch devices, respectively. Solid and hollow symbols represent measured AE current and voltage, respectively, and dashed curves show fitted model predictions used as visual guidance. The AE response increases approximately linearly with input power over the measured range, consistent with analytical predictions. The horizontal purple line indicates the DC readout noise floor. A peak AE current of 61.17 μA and AE voltage of 225.43 mV is recorded at ${P}_{{{\rm{in}}}}=\,20{{\rm{dBm}}}$ for the 10 μm pitch device.

Next, to examine the dependence of AE response on input power, both AE current and AE voltage were measured as a function of RF input power at the center frequency of the corresponding SAW passband. This is shown in Fig. 4, alongside theoretical predictions fitted to Eq. (3). For theoretical calculations, ${K}^{2}=0.48$ and ${v}_{{{\rm{SAW}}}}=1962$ m/s were obtained experimentally from SAW delay line characterizations and time-of-flight measurements (Supplementary Fig. 2). For AE current calculations, SAW intensity was taken as the drive power reduced by half of the end-to-end insertion loss^24^. At an input power of 20 dBm, for the 10 μm pitch device, maximum AE current and voltage of 61.17 μA and 225.43 mV are measured, respectively (Fig. 4).

**Fig. 5:**
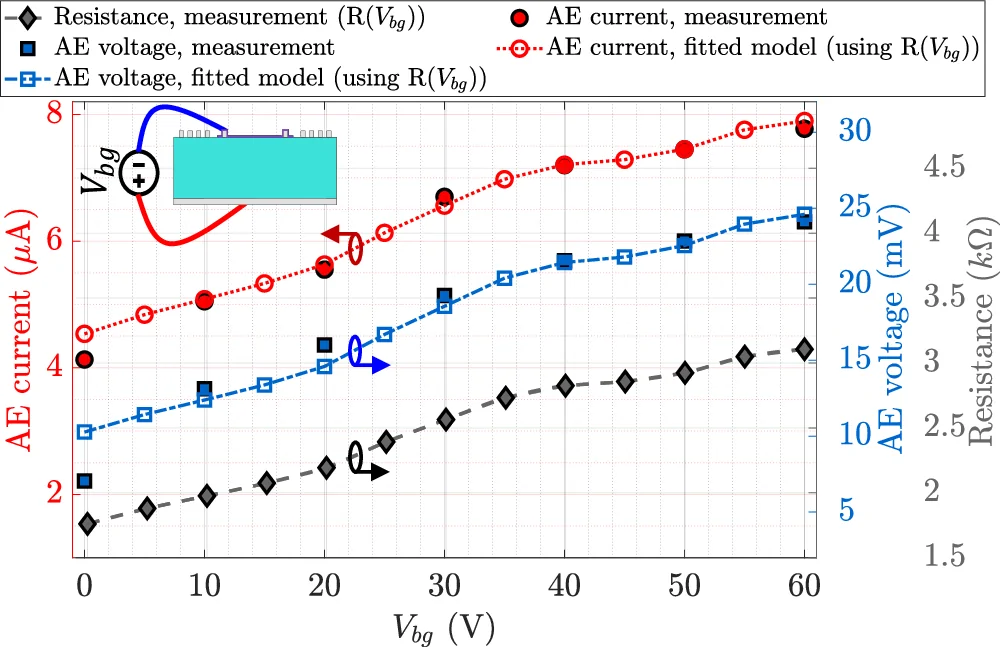
Gate tunable acoustoelectric response. Gate-tunable AE response and graphene resistance for a device with FP = 10 µm and *L* = 300 µm, where FP is the IDT finger pitch, and L is the length of the graphene coupling region along the surface acoustic wave propagation path. Measured graphene resistance $R({V}_{{{\rm{bg}}}})$ (gray diamonds) plotted together with the corresponding AE current ${I}_{{{\rm{AE}}}}$ (solid red circles) and AE voltage ${V}_{{{\rm{AE}}}}$ (solid blue squares) as a function of back-gate voltage ${V}_{{{\rm{bg}}}}$ at ${P}_{{{\rm{in}}}}=10$ dBm. Increasing ${V}_{{{\rm{bg}}}}$ monotonically increases $R\left({V}_{{{\rm{bg}}}}\right)$ and correspondingly enhances both ${I}_{{AE}}$ and ${V}_{{{\rm{AE}}}}$. Hollow curves show fitted model predictions for ${I}_{{{\rm{AE}}}}$ and ${V}_{{{\rm{AE}}}}$ calculated using the experimentally measured $R({V}_{{{\rm{bg}}}})$ at each bias point. Dashed curves are for visual guidance. The inset illustrates the back-gate measurement configuration, where a voltage is applied between the backside electrode and graphene to tune the graphene sheet conductivity.

**Fig. 6:**
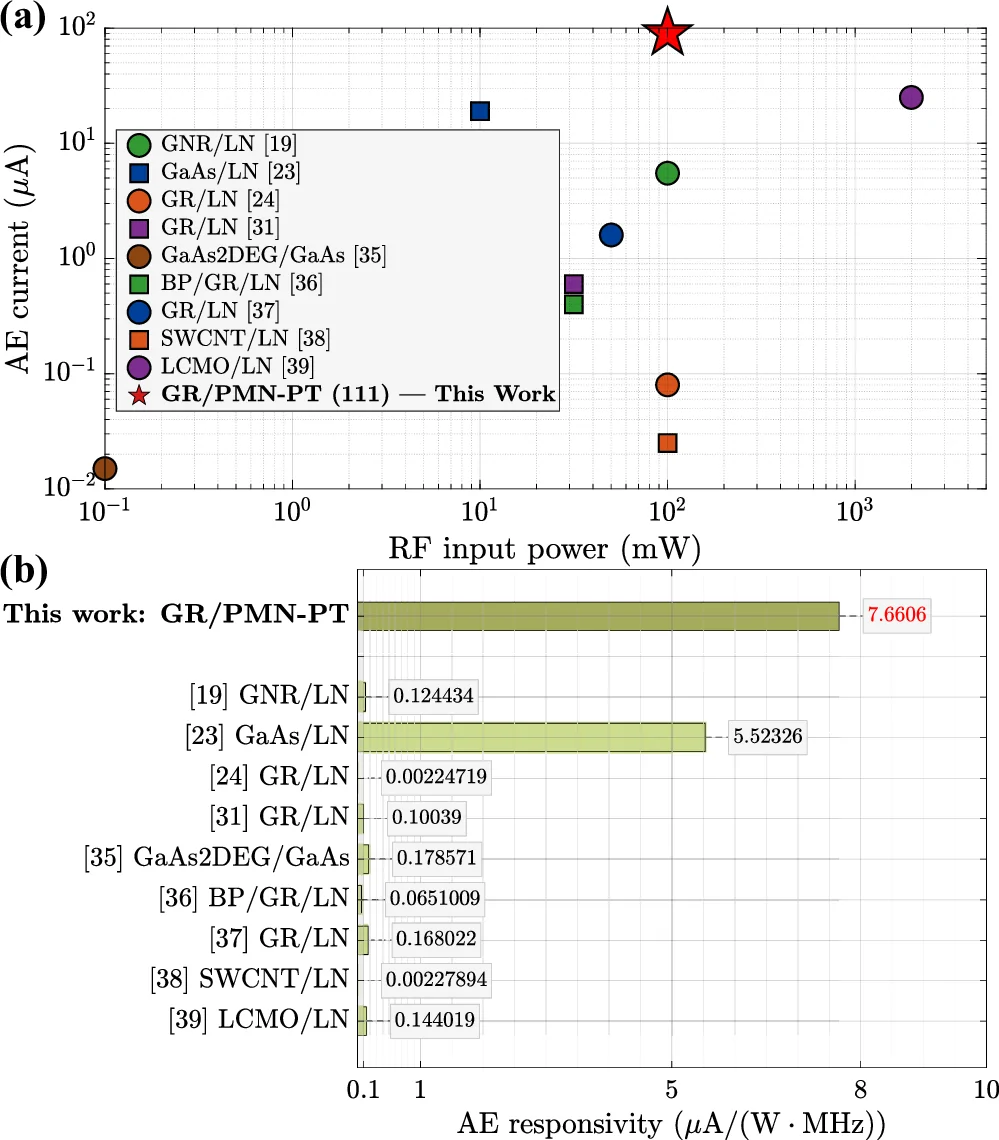
Benchmark comparison of acoustoelectric current generation and AE responsivity. **a** Survey of the highest AE current versus maximum RF power reported across SAW platforms^19,23,24,31,35–39^ (red star = this work). **b** Comparison of AE responsivity, ${{{{\mathscr{R}}}}_{{{\rm{AE}}}}=I}_{{{\rm{AE}}}}/({P}_{{{\rm{in}}}}\cdot f)$, for the same platforms. Here, ${{{\mathscr{R}}}}_{{{\rm{AE}}}}$ is defined as the AE current normalized to RF input power (${P}_{{{\rm{in}}}}$) and operating frequency (f). ${{{\mathscr{R}}}}_{{{\rm{AE}}}}$ is introduced here as a figure of merit for cross-platform benchmarking. This metric enables comparison between AE platforms operating at different frequencies and input powers. The measured AE current and AE responsivity outperform previously reported platforms.

Since the graphene film conductivity is higher than the characteristic conductivity (${\sigma }_{\square } > {\sigma }_{{{\rm{m}}}}$), an electric field is applied to modulate the graphene conductivity and reduce ${\sigma }_{\square }$ toward ${\sigma }_{{{\rm{m}}}}$ in order to intensify the AE attenuation, and subsequently boost the AE current and voltage (Eq. 3). This is realized by a DC bias on the backside PMN–PT electrode serving as a global back-gate to shift the graphene Fermi level and sheet carrier density without additional scattering. Figure 5 shows that increasing ${V}_{{{\rm{bg}}}}$ monotonically increases the graphene resistance $R({V}_{{{\rm{bg}}}})$, improving conductivity matching (${\sigma }_{{{\square }}}\to {\sigma }_{{{\rm{m}}}}$) and thereby boosting AE current and voltage. The results correspond to a 10 µm pitch device at ${P}_{{{\rm{in}}}}=$ 10 dBm and are consistent with theoretical predictions fitted to Eqs. (2) and (3) using the measured $R({V}_{{{\rm{bg}}}})$ at each gate voltage point. Strictly, the acoustoelectric model should use the sheet resistance at the SAW frequency; however, the DC resistance is used here as a valid approximation to capture the gate-dependent conductivity trend, consistent with common practice in the literature. Said gate-tunable trend is reproduced across six devices with different pitches and delay lengths (Supplementary Fig. 1). Additionally, no sign change was observed in the AE signal as ${V}_{{{\rm{bg}}}}$ was increased to 80 V, highlighting operation in the p-type region and not crossing the Dirac point, where the SAW-dragged carriers switch from holes to electrons^31^. At 20 dBm input power and for ${V}_{{{\rm{bg}}}}\,$ = 50 V, a giant AE current of 90.39 μA and AE voltage of 341.72 mV was achieved for the best-performing device, surpassing prior benchmarks (Fig. 6a). Furthermore, to enable a fair comparison across platforms operating at different RF powers and frequencies, the AE responsivity is reported (Fig. 6b), due to the linear scaling of ${I}_{{{\rm{AE}}}}$ with input power and frequency in the small-signal regime. Specifically, compared to the best-performing platform, reported in ref. ^23^, this work achieves ~1.38× higher output, which could be further improved to 4× via IDT design optimization while avoiding complex and resource-intensive integration of III-V thin films on lithium niobate (LiNbO_3_). Additional width-normalized and full-aperture-projected comparisons are provided in Supplementary Fig. 4, further supporting the strong AE response of the graphene-on-PMN–PT platform across different normalization approaches.

## Discussion

Giant acoustoelectric current generation in a SAW delay line platform based on a monolayer graphene film integrated onto a (111)-cut PMN–PT substrate is demonstrated. By leveraging the strong electromechanical coupling of PMN–PT and the high carrier mobility of graphene, the platform exhibits robust AE response within the SAW passband. Experimental measurements confirm the frequency selectivity and power-dependent behavior of the AE signal, in agreement with analytical predictions. Notably, AE currents up to 61.17 μA and voltages up to 225.43 mV are achieved under 20 dBm RF input power without back-gate voltage. Furthermore, by applying a back-gate voltage, this platform allows for electrostatically modulating graphene sheet conductivity while maintaining carrier mobility unchanged. This enhances the AE current to 90.39 μA and voltage to 341.72 mV for a 50 V gate voltage. These experimentally confirmed values surpass existing benchmarks for AE current generation. AE responsivity is introduced here as a figure of merit to enable more direct comparison across platforms reported at different input powers and frequencies. Using this metric, the present platform reaches 7.66 µA/(W·﻿MHz), which is ~1.38× higher than the best previously reported value^23^, further establishing the graphene-on-PMN–PT SAW platform as a promising candidate for next-generation on-chip charge transport and sensing technologies.

## Methods

### Fabrication

A 100 nm molybdenum (Mo) layer is sputtered on both sides for IDT electrodes and poling (Fig. 2a). Poling is essential for activating the piezoelectric response of PMN–PT by aligning ferroelectric domains^32^. The domain structure and polarization of (111)-cut PMN–PT single crystals are highly sensitive to poling conditions^33^, necessitating appropriate poling to optimize dielectric and piezoelectric properties by balancing domain alignment, thermal stress, and mitigating the risk of dielectric breakdown^34^. It is worth noting that after poling, due to PMN–PT’s low Curie temperature (~100 °C), maintaining a low thermal budget during fabrication is essential. The IDTs are defined by lithography (Fig. 2b) and reactive ion etching (RIE) (Fig. 2c). Next, the monolayer graphene is wet transferred onto the PMN–PT substrate at room temperature (Fig. 2d and further details in “Methods,” section “Graphene transfer process”). Following graphene transfer, a second lithography step is carried out to define the AE coupling region on graphene via oxygen plasma RIE (Fig. 2e, f).

### Graphene transfer process

A polymer-free wet transfer technique was employed for graphene transfer. Chemical vapor deposition (CVD)-grown graphene on copper foil was cut to the desired dimensions for the target substrate and placed on the surface of a 0.1 M ammonium persulfate solution for copper etching, with the sample centered using a polyethylene terephthalate (PET) retainer. After 2–3 h of etching, a free-floating graphene film was obtained within the central opening of the PET retainer. The etching solution was subsequently replaced several times with deionized (DI) water to remove residual contaminants. The target substrate was then positioned directly beneath the graphene film, and the DI water was slowly drained through the bottom-side outlet to laminate the graphene onto the substrate. Finally, the graphene-transferred substrate was dried under a gentle flow of nitrogen gas to remove residual water.

## Supplementary information

Supplementary Information

Transparent Peer Review file

## Data Availability

Relevant data supporting the key findings of this study are available within the article and the Supplementary Information file. All raw data generated during the current study are available from the corresponding authors upon request.
