# Supplementary material for: Observation of giant acoustoelectric currents in graphene on lead magnesium niobate–lead titanate
Source: Nat Commun. 2026 Jul 10;17:8932. doi: 10.1038/s41467-026-74813-3 (PMC13503809; doi:10.1038/s41467-026-74813-3)
Supplement: Supplementary file 1 [file 41467_2026_74813_MOESM1_ESM.pdf]

Supplementary Information
"Observation of Giant Acoustoelectric Currents in Graphene on Lead Magnesium Niobate–Lead Titanate"
H. Atashbar et al.

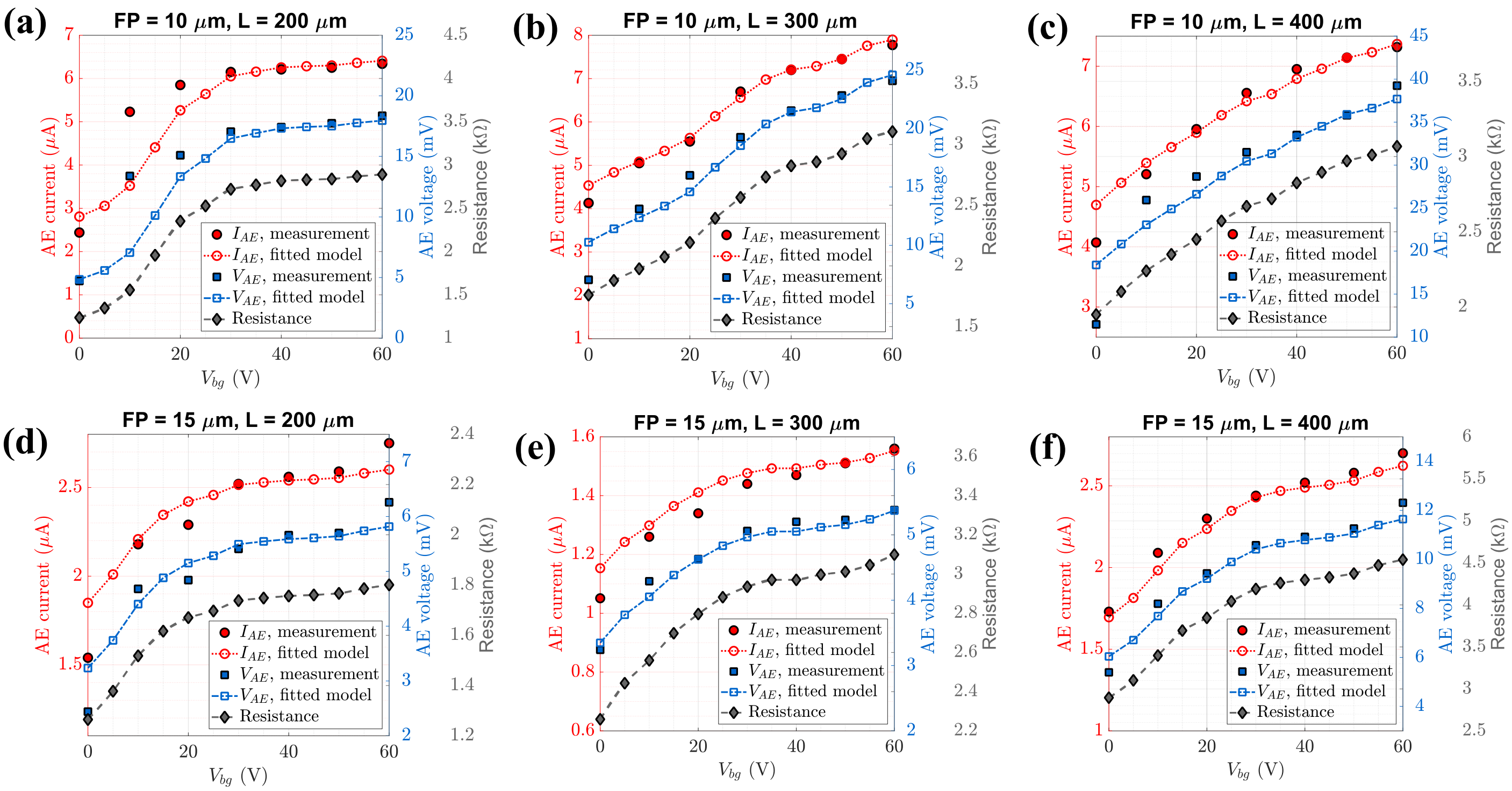

Supplementary Figure 1. | Back-gate tuning of graphene resistance and AE response. Graphene resistance, AE current, and AE voltage versus gate voltage for devices with finger pitch FP = 10 µm **(a–c)** and FP = 15 µm **(d–f)**, and delay lengths L = 200, 300, and 400 µm. Each panel shows measured data and fitted theoretical curves for AE current and voltage ($P_{\text{in}} = 10$ dBm), along with measured graphene resistance. Increasing gate voltage raises graphene resistance, indicating reduced conductivity as the device is tuned toward the charge-neutrality (Dirac) point; the gate voltage increase up to 60 V suggests the Dirac point lies beyond the measured range ($V_{\text{Dirac}} >> 60$ V). Dashed curves are for visual guidance.

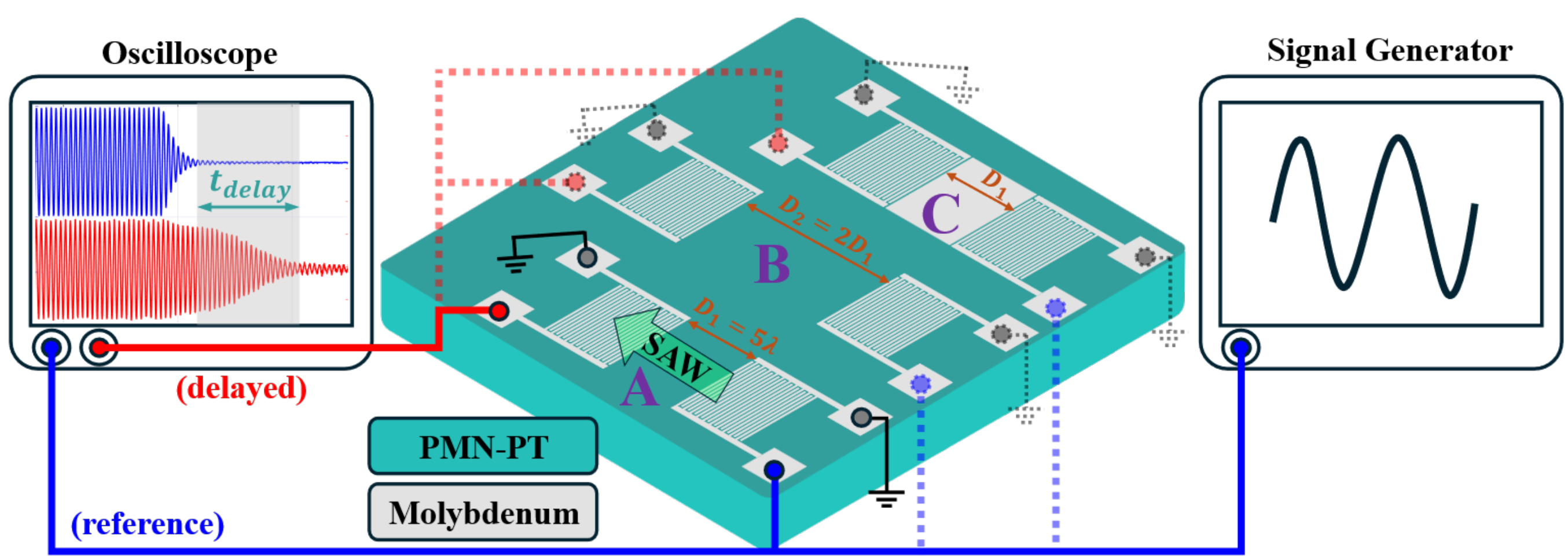

Supplementary Figure 2. | Time-of-flight (ToF) method for extracting SAW velocity and electromechanical coupling (K^2) in SAW delay lines. Three nominally identical IDT configurations are used: (A) a free-surface delay line with gap length $D_1 = 5\lambda$, (B) a free-surface delay line with doubled gap $D_2 = 10\lambda$, and (C) a delay line identical to (A) but with continuous metallization in the gap to form a short-circuited (metallized) surface. All devices use IDTs with finger pitch $FP = 10\ \mu m$, 10 finger pairs, and a 50% metallization ratio, yielding $\lambda = 2FP$; the aperture is $W = 10\lambda$. Transit times t_1, t_2, and t_3 are obtained from the measured input-output delays of (A), (B), and (C), respectively, and are used to compute the free-surface and metallized-surface velocities (v_{free} and v_{metal}) and, subsequently, K^2 via Eqs. (S1)-(S4).

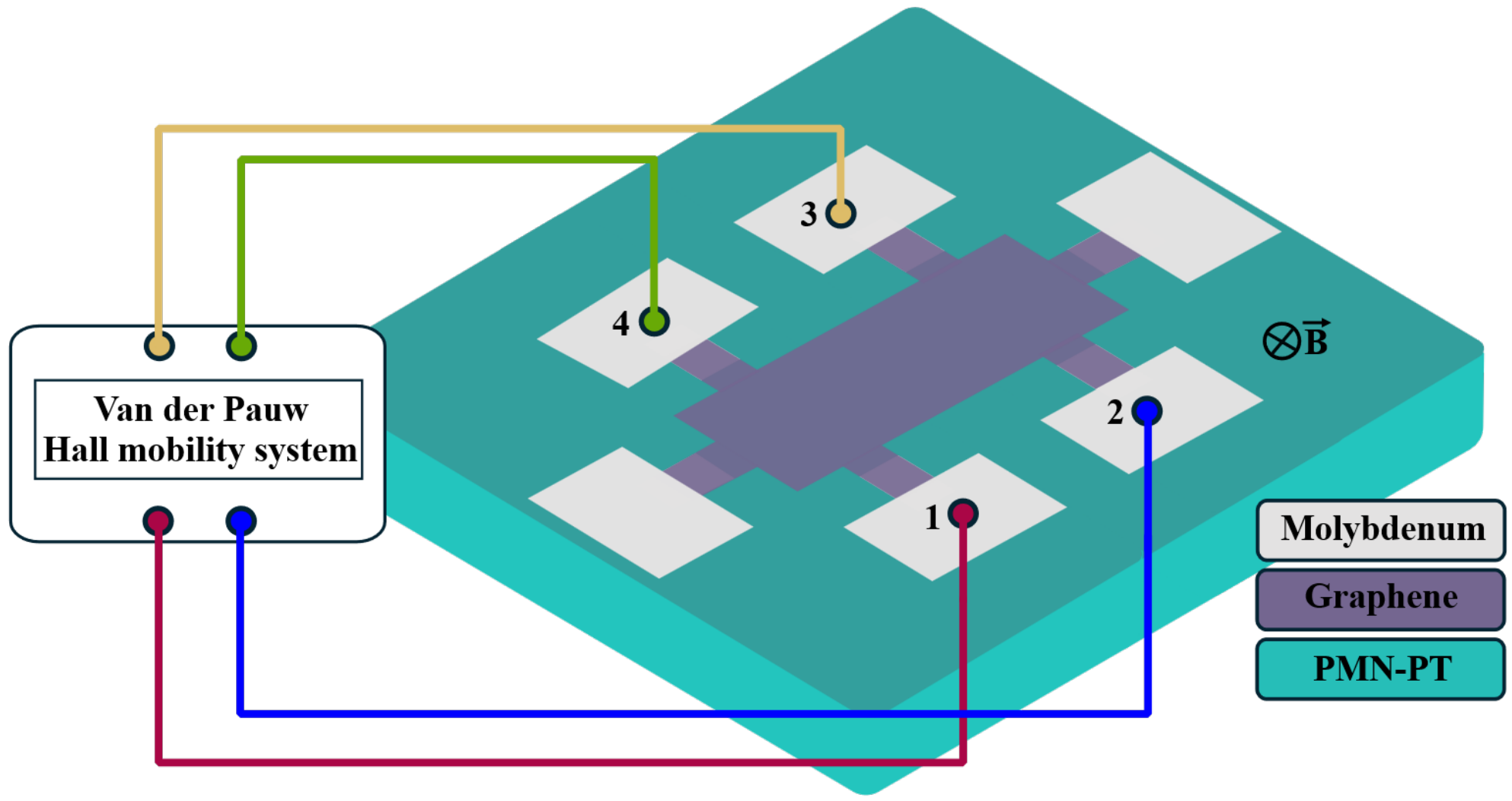

Supplementary Figure 3. | Van der Pauw Hall mobility measurement setup. Schematic of the Hall characterization configuration used to extract the graphene sheet carrier density and mobility using a Van der Pauw Hall system. Four contacts (1-4) are connected to the measurement unit while an out-of-plane magnetic field B is applied. The measured stack consists of patterned graphene on molybdenum contacts on a (111)-cut PMN-PT substrate.

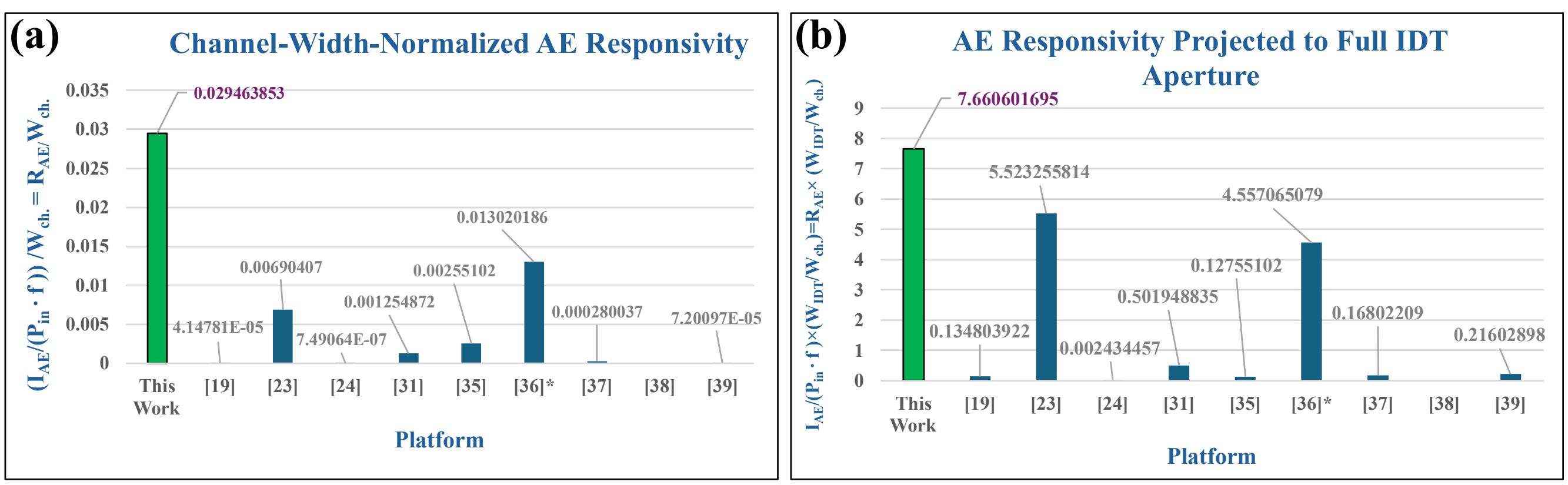

Supplementary Figure 4. | Width-normalized benchmark comparison of AE responsivity ($\mathcal{R}_{AE}$). Reference numbers correspond to the numbering used in the main manuscript reference list. **(a)** AE responsivity normalized by the active 2D channel width ($\mathcal{R}_{AE,W}$), defined as $I_{AE}/(P_{in} \cdot f \cdot W_{ch})$, where I_{AE} is the measured AE current, P_{in} is the RF input power, f is the operating frequency, and W_{ch} is the active channel width. This metric removes the direct scaling of $\mathcal{R}_{AE}$ with channel width. **(b)** Full-aperture-projected $\mathcal{R}_{AE}$, calculated as $I_{AE}W_{IDT}/(P_{in} \cdot f \cdot W_{ch})$, where W_{IDT} is the IDT aperture width. This projection estimates the expected $\mathcal{R}_{AE}$ if each 2D conducting channel extended across the full IDT aperture. Ref. [36] (in the manuscript) is marked here with an asterisk because the BP/hBN/GR/LN platform used an exfoliated BP channel with an effective width of about 5 µm within a 350 µm IDT aperture; therefore, its full-aperture-projected value should be interpreted as an optimistic upper-bound estimate.

Supplementary Note 1. Back-gate tuning of graphene resistance and AE response

Supplementary Fig. 1 shows that applying a back-gate voltage can enhance AE current and voltage by increasing the graphene resistance (i.e., reducing sheet conductivity), which results in a closer conductivity-matching between the graphene film and the SAW electric field. This is performed on six geometrically different devices with IDT pitches of 10 and 15 µm, and delay lengths of 200, 300, and 400 µm. Over gate voltages of 0-60 V, the graphene resistance increases monotonically, indicating the device is still approaching the charge-neutrality (Dirac) point rather than crossing it (V_{Dirac} >> 60 V); beyond the Dirac point, the resistance is expected to decrease. Importantly, after each V_{bg} step we observe a slow relaxation of $R(V_{bg})$ toward its steady-state value; therefore, for all gate-dependent datasets reported here, we wait at each bias until R stabilizes (typically ~ 30 min per point) before recording R, I_{AE}, and V_{AE} (measured at $P_{in} = 10$ dBm). Such long settling is consistent with charge trapping/detrapping in graphene/PMN-PT interface reported in [1]. Although one device was tested up to 80 V and the resistance was still increasing, we limited V_{bg} to 60 V to avoid applying a high electric field opposite to the poling direction, which could risk depoling or device damage.

Supplementary Note 2. SAW velocity and K^2 extraction using time-of-flight measurements

To quantify coupling (K^2) in SAW delay lines, the velocity-based extraction method was used from acoustic phase velocities on an electrically free surface (v_{free}) and a short-circuited metallized surface (v_{metal}) given by [2]:

$$K^2 = (v_{\text{free}}{}^2 - v_{\text{metal}}{}^2)/v_{\text{free}}{}^2 \,. \quad \text{(S1)}$$

For obtaining coupling, three nominally identical IDT configurations were implemented as illustrated in Supplementary Fig. 2: a free-surface delay line with gap length $D_1 = 5\lambda$ (A); a free-surface device with a doubled gap $D_2 = 10\lambda$ (B); and a device identical to (A) but with a continuous metallization in the gap to realize a short-circuited surface (C). The measured devices comprise IDTs with finger pitch (FP) $FP = 10$ µm, with 10 finger pairs of a ~50% metallization ratio, yielding $\lambda = 2FP$. The IDT aperture is $W = 10\lambda$ for all devices. Transit times are attained from the measured delay between input and output for the three structures. Let t_1, t_2, and t_3 denote the delays of the devices with gap lengths D_1 (free surface: A), $D_2 = 2D_1$ (free surface: B), and $D_3 = D_1$ (metallized: C), respectively. The free-surface phase velocity follows from the differential delay between the two free-surface paths,

$$v_{\text{free}} = \frac{D_1}{t_2 - t_1}, \quad \text{(S2)}$$

and the metallized-surface velocity is,

$$v_{\text{metal}} = \frac{D_1}{t_2 + t_3 - 2t_1} \,. \quad \text{(S3)}$$

Using Eq. (S1), the electromechanical coupling of ~48% is then obtained from the equation below,

$$K^2 = 1 - \left(\frac{t_2 - t_1}{t_2 + t_3 - 2t_1}\right)^2 . \quad \text{(S4)}$$

Supplementary Table 1 presents the measured time-of-flight results and the extracted coupling.

Supplementary Table 1. Measured time-of-flight results and coupling for Graphene/PMN-PT SAW delay line.

t_1 (ns)	t_2 (ns)	t_3 (ns)	v_{free} (m/s)	v_{metal} (m/s)	Coupling (%)
184	234.96	204	1962.32	1409.25	48.42

Supplementary Note 3. Hall and Van der Pauw characterization of graphene

Using a Van der Pauw Hall system (MMR Technologies), we measured a room-temperature Hall mobility of $\mu = 400.4$ cm^2/(V·s) for the graphene film, as measured using the setup shown in Supplementary Fig. 3 and summarized in Supplementary Table 2.

Supplementary Table 2. Hall/Van der Pauw electrical characterization of graphene. Extracted sheet resistivity, Hall coefficient, sheet carrier density, and Hall mobility for the measured graphene sample at room temperature.

Sheet resistivity (Ω/□)	Hall coefficient (cm^3/C)	Sheet carrier density (cm^{-2})	Mobility (cm^2/(V·s))
1188.84	0.0476	1.31×10^{13}	400.4

Supplementary Note 4. Benchmarking AE response beyond channel width

To examine whether the large AE response reported in this work arises primarily from the larger lateral interaction width, we benchmarked the reported 2-D AE platforms using two width-aware metrics. In Supplementary Fig. 4a, the AE responsivity ($\mathcal{R}_{\text{AE}}$) is normalized by the active 2-D channel width as $I_{\text{AE}}/(P_{\text{in}} \cdot f \cdot W_{\text{ch}})$, where I_{AE} is the measured AE current, P_{in} is the RF input power, f is the operating frequency, and W_{ch} is the active conducting channel width. This width-normalized metric removes the direct contribution of channel width to the measured AE current and therefore provides a more geometry-aware comparison across platforms. Under this normalization, the graphene/PMN-PT platform reaches 0.0295 µA/(W·Hz·m), which is the highest value among the compared systems and more than 4.2× larger than Ref. [23] in the manuscript.

Supplementary Fig. 4b presents a full-aperture-projected AE responsivity, calculated as $I_{\text{AE}} \cdot W_{IDT}/(P_{\text{in}} \cdot f \cdot W_{\text{ch}})$, where W_{IDT} is the IDT aperture width. This metric estimates the AE responsivity that would be expected if each 2-D conducting channel

were extended to cover the full acoustic aperture. Therefore, for platforms based on narrow exfoliated flakes, this value should be interpreted as an optimistic upper-bound projection rather than an experimentally demonstrated full-aperture response. Even under this projected comparison, the graphene/PMN-PT platform remains the highest-performing system.

Ref. [36] in the manuscript is marked here with an asterisk because it reports a BP/hBN/GR/LN heterostructure based on exfoliated black phosphorus. In that work, the reported BP channel width is approximately 5 µm, whereas the IDT aperture width is 350 µm. Because exfoliated BP flakes are typically small and irregularly shaped, extending the same BP/hBN/GR heterostructure uniformly across the full IDT aperture is not straightforward. Accordingly, the full-aperture-projected value for Ref. [36] in the manuscript is included for completeness but should be regarded as an optimistic upper-bound estimate rather than a demonstrated full-aperture device value.

Overall, these width-aware comparisons show that the large AE current and responsivity in this work are not solely a consequence of the larger interaction width. Instead, the graphene/PMN-PT platform combines a strong width-normalized AE response with practical full-aperture integration of graphene across the IDT aperture.

Supplementary References

[1] Jie, W. & Hao, J. Time-dependent transport characteristics of graphene tuned by ferroelectric polarization and interface charge trapping. *Nanoscale* **10**, 328–335 (2018). https://doi.org/10.1039/C7NR06485C.

[2] Mansoorzare, H. & Abdolvand, R. Micromachined heterostructured Lamb mode waveguides for acoustoelectric signal processing. *IEEE Trans. Microw. Theory Tech.* **70**, 5195–5204 (2022). https://doi.org/10.1109/TMTT.2022.3194723.
