# Supplementary material for: Observation of giant acoustoelectric currents in graphene on lead magnesium niobate–lead titanate
Source: Nat Commun. 2026 Jul 10;17:8932. doi: 10.1038/s41467-026-74813-3 (PMC13503809; doi:10.1038/s41467-026-74813-3)
Supplement: Supplementary file 2 [file 41467_2026_74813_MOESM2_ESM.pdf]

nature portfolio

Peer Review File

Observation of Giant Acoustoelectric Currents in Graphene on Lead Magnesium Niobate–Lead Titanate

Corresponding Author: Mr Hamed Atashbar

Parts of this Peer Review File have been redacted as indicated to remove third-party material.

This file contains all reviewer reports in order by version, followed by all author rebuttals in order by version.

Version 0:

Reviewer comments:

Reviewer #1

(Remarks to the Author)
The paper demonstrates 'giant' acoustoelectric (AE) currents generated by surface acoustic waves (SAWs) in a delay-line where monolayer graphene is placed in the propagation path on a bulk (111) PMNPT single-crystal substrate. The aim is to maximize AE current at room temperature for acoustic charge transport, sensing and RF signal-processing.
The platform is competently engineered and PMNPT is a great choice of piezoelectric; the experimental execution is careful in several places (e.g., time-gated S_21 to isolate SAW, which every engineer appreciates).

That said, the manuscript's focus is device integration and performance scaling, not new physics. The AE mechanism, the graphene gating response, and the relaxation-model description are well-established. The work does not uncover a new phenomenon, regime, or physical insight beyond demonstrating higher AE magnitudes by combining graphene with a high-K substrate and tuning \sigma_\square.

In my view this makes the paper a better fit for an applied/engineering venue that prioritizes SAW device platforms and system-level performance over advances in physical understanding. Suitable outlets might include IEEE TUFFC, Sensors & Actuators A, Microsystems & Nanoengineering, or IEEE Electron Device Letters. To be clear and constructive: the device results are promising for applications, and the manuscript can have real impact with readers focused on SAW/AE engineering.

Hence, while the experimental platform is solid and the PMN-PT choice is technically astute, the manuscript does not advance physical understanding of the acoustoelectric effect or graphene transport. Rather, it demonstrates incremental performance gains via materials/platform engineering and well-known conductivity matching. For a physics journal, that is not enough: I therefore recommend rejection on scope. I encourage the authors to reframe for an engineering-focused journal, where the careful integration and promising AE figures will likely be appreciated and cited.

Reviewer #2

(Remarks to the Author)
The authors report the observation of giant acoustoelectric (AE) currents in a graphene sample integrated on a novel PMN-PT SAW platform via an etching and wet-transfer process with low thermal budget. By leveraging the high carrier mobility of graphene and the exceptional electromechanical coupling strength of (111)-cut PMN-PT, they achieve a high AE current up to 90 µA. The primary innovation of this work lies in the adoption of a new SAW substrate material, while the physics discussion still lacks depth and original insight. In its current form, I consider the manuscript to be highly suitable for a more specified applied physics journal. Some of this may be related to presentation and interpretation. Below is my detailed comment for further strengthen the manuscript:
1, In principle, one could always obtain larger AE currents simply by increasing the incident RF power. Therefore, comparing the absolute AE current alone is not physically meaningful. For practical acousto-electronic device development, the key metric is the AE generation efficiency, i.e., the AE current per unit input power (equivalent to the slope of Fig. 4). I suggest

benchmarking this efficiency against previous studies.
2, Another factor limiting AE current/voltage is the saturation of the AE effect at high SAW intensities due to limited carrier density and potential nonlinear effects (see APL 73, 2128 (1998); PRL 82, 2171 (1999); Nano Lett. 25, 14082 (2025)). Have the authors observed any nonlinear behavior in their experiments? If not, they should discuss why the AE current/voltage remains linearly dependent on input RF power even up to +20 dBm.
3, The authors state K2=0.48 and v_SAW=1962 m/s for (111)-cut PMN-PT based on characterization of SAW delay lines. Please clarify whether these values are experimentally measured or taken from prior literature. Since the electromechanical coupling coefficient is the key parameter responsible for the giant AE effect, I strongly recommend including both experimental and calculation details—possibly in the Supplementary Information—to ensure scientific rigor.
4, The manuscript only reports resistance values of 1.3-2.3 kΩ for the graphene sample, without showing any transfer curve. I suggest including the transfer characteristics and other basic electrical measurements to allow more intuitive comparison with the AE performance.
5, At the end of Section II, the statement "It is worth noting that charge carrier mobility has been shown to remain independent of back-gate voltage [28]" is inaccurate, as Ref. 28 shows clear deviations near the charge neutrality point. In Section IV, the mobility value is directly adopted from the reference. I recommend including experimental mobility extraction for the actual samples to support the analysis. Does the measured mobility quantitatively fit Equation 3, given that the attenuation and sound intensity are experimentally accessible?
6, How is the theoretical curve in Fig. 5 obtained? Specifically, how were the capacitance Cg and mobility μ values in Equation 4 determined? More details should be provided.
7, Some numerical values in the data appear overly precise or coincidental—for example, the reported 50 µA and 90 µA in the captions of Fig. 4 and Fig. 5. What is the measurement resolution? Were the values exactly 50 and 90 µA? Please clarify.
8, There seems to be a typo in the statement "Maximum AE interaction occurs when the two conductivities match (i.e., □=σ?)".

Reviewer #3

(Remarks to the Author)
I co-reviewed this manuscript with one of the reviewers who provided the listed reports. This is part of the Nature Communications initiative to facilitate training in peer review and to provide appropriate recognition for Early Career Researchers who co-review manuscripts.

Reviewer #4

(Remarks to the Author)
• What are the noteworthy results?
- The work demonstrates record-high acoustoelectric current generation, up to 90 µA at 20 dBm, using a graphene-on-(111) PMN-PT SAW platform, confirming efficient acoustic charge transport and highlighting strong potential for sensing, quantum, and signal-processing applications.
• Will the work be of significance to the field and related fields? How does it compare to the established literature? If the work is not original, please provide relevant references.
- Yes, the work is significant, demonstrating record-high acoustoelectric current generation in a graphene–PMN-PT SAW platform. It surpasses prior reports using materials like GaAs or $LiNbO_3$, which achieved only nanoampere to low-microampere currents, thus representing a clear advancement in acoustic charge transport research.
• Does the work support the conclusions and claims, or is additional evidence needed?
- yes
• Are there any flaws in the data analysis, interpretation and conclusions? Do these prohibit publication or require revision?
- No
• Is the methodology sound? Does the work meet the expected standards in your field?
- yes
• Is there enough detail provided in the methods for the work to be reproduced?
- On page 2, Section II, please clarify what the symbol σ represents, is it referring to σ_m (metal conductivity) or something else?
- please provide further detail on the time gating criteria of the SAW data.
- It would be helpful to briefly explain the graphene transfer process shown in Figure 2, so that readers unfamiliar with the method can follow more easily.
- It would also be valuable to comment on the polarization direction of PMN-PT relative to Vbg and discuss its potential impact on graphene conductivity.

Version 1:

Reviewer comments:

Reviewer #2

(Remarks to the Author)
We thank the authors for their efforts in addressing the previous round of comments. Additional measurements have been performed, and the manuscript has been revised to provide further clarification of the experimental procedures. The device

characterization is now more complete, and the physical discussions have been expanded. Nevertheless, after careful consideration of the revised manuscript and the authors responses, I remain somewhat disappointed with the current version.
1, Figure 6 compares the device performance with other studies. After normalizing by input power, the reported results do not demonstrate a significant improvement than that of Ref. 22 (GaAs/LN).
2, Compared to other works on the acoustoelectric effect in 2D materials, the apparent enhancement in acoustoelectric response appears to stem largely from the unusually large sample size. In this work, the 2D material fully spans the IDT aperture, whereas most prior studies employ flakes that are typically an order of magnitude smaller than the IDT width. This geometric advantage raises concerns as to whether the observed signal truly reflects intrinsic material properties or is primarily a consequence of the increased interaction area.
3, In Fig. 3, the authors present a Hall bar device but extract mobility using only the four central contacts via the van der Pauw method. This approach is unconventional and potentially problematic: it neglects the influence of the source/drain metal contacts (not used in van der Pauw method) and disregards the complex current distribution inherent to complex Hall bar geometries. These oversights can significantly increase measurement uncertainty and introduce systematic errors. Why was a standard six-terminal Hall measurement not employed instead? Or they can just use the van der Pauw method on a standard square device instead on the Hall bar device.
4, The sheet conductance in Equation 2 actually corresponds to the conductance at SAW frequency. However, the authors substitute experimentally measured DC resistance directly into this expression. Moreover, they totally neglected the contact resistance.
5, The authors report requiring ~30 minutes of relaxation at each gate voltage before stable transport data can be acquired. Such a slow response is difficult to reconcile even with known charge trapping mechanisms in 2D heterostructures.
6, Did the authors monitor gate leakage currents? Could Joule heating due to gate leakage contribute to the observed drift or instability? If substantial gate leakage exists, it could also lead to large DC currents flowing through the channel, potentially masking or distorting the true acoustoelectric signal. We suggest the authors: (i) test negative gate biases, and (ii) consider modulating the SAW at low frequency and using lock-in detection to isolate the genuine acoustoelectric response from background drift or thermal artifacts.

Reviewer #3

(Remarks to the Author)
I co-reviewed this manuscript with one of the reviewers who provided the listed reports. This is part of the Nature Communications initiative to facilitate training in peer review and to provide appropriate recognition for Early Career Researchers who co-review manuscripts.

Version 2:

Reviewer comments:

Reviewer #2

(Remarks to the Author)
I thank the authors for their responses to previous comments. I find the clarification regarding the AE response and the width-normalized benchmarking analysis to be satisfactory. Regarding the use of DC resistance in the model, while I understand that this is a common practice in the literature, I suggest the authors explicitly state in the main text that the rigorous approach should utilize the AC resistance at the SAW frequency, and that the use of DC values serves as a valid approximation. Additionally, I recommend briefly discussing the rationale for the ~30-min relaxation time observed in graphene on PMN-PT.
Overall, the manuscript has improved substantially in both completeness and reliability, and I recommend it for publication.

Open Access This Peer Review File is licensed under a Creative Commons Attribution 4.0 International License, which permits use, sharing, adaptation, distribution and reproduction in any medium or format, as long as you give appropriate credit to the original author(s) and the source, provide a link to the Creative Commons license, and indicate if changes were made.
In cases where reviewers are anonymous, credit should be given to 'Anonymous Referee' and the source.
The images or other third party material in this Peer Review File are included in the article's Creative Commons license, unless indicated otherwise in a credit line to the material. If material is not included in the article's Creative Commons license and your intended use is not permitted by statutory regulation or exceeds the permitted use, you will need to obtain permission directly from the copyright holder.
To view a copy of this license, visit https://creativecommons.org/licenses/by/4.0/

We would like to thank the reviewers for their valuable comments and feedback. Below, the questions and comments of each reviewer are listed along with our response.

Reviewer 1

The paper demonstrates 'giant' acoustoelectric (AE) currents generated by surface acoustic waves (SAWs) in a delay-line where monolayer graphene is placed in the propagation path on a bulk (111) PMNPT single-crystal substrate. The aim is to maximize AE current at room temperature for acoustic charge transport, sensing and RF signal-processing.

The platform is competently engineered and PMNPT is a great choice of piezoelectric; the experimental execution is careful in several places (e.g., time-gated S_21 to isolate SAW, which every engineer appreciates).

That said, the manuscript's focus is device integration and performance scaling, not new physics. The AE mechanism, the graphene gating response, and the relaxation-model description are well-established. The work does not uncover a new phenomenon, regime, or physical insight beyond demonstrating higher AE magnitudes by combining graphene with a high-K substrate and tuning \sigma_\square.

In my view this makes the paper a better fit for an applied/engineering venue that prioritizes SAW device platforms and system-level performance over advances in physical understanding. Suitable outlets might include IEEE TUFFC, Sensors & Actuators A, Microsystems & Nanoengineering, or IEEE Electron Device Letters. To be clear and constructive: the device results are promising for applications, and the manuscript can have real impact with readers focused on SAW/AE engineering.

Hence, while the experimental platform is solid and the PMN-PT choice is technically astute, the manuscript does not advance physical understanding of the acoustoelectric effect or graphene transport. Rather, it demonstrates incremental performance gains via materials/platform engineering and well-known conductivity matching. For a physics journal, that is not enough: I therefore recommend rejection on scope. I encourage the authors to reframe for an engineering-focused journal, where the careful integration and promising AE figures will likely be appreciated and cited.

Answer to the reviewer:

We sincerely thank the reviewer for acknowledging the quality of the developed platform. We agree that the central contribution of our work is not discovery of a new acoustoelectric mechanism, rather the demonstration of a substantial performance/functionality improvement in graphene-based acoustoelectrics by combining (1) high mobility graphene film integration with (2) a record high electromechanical-coupling SAW platform. This framing is aligned with *Nature Communications*' stated interest in application-driven 2D technologies where performance optimization and benchmarking are critical criteria for assessing significance.

Concretely, the technological advances demonstrated here include:

- **Record-high acoustoelectric (AE) current and AE responsivity ($\mathcal{R}_{AE}$) in a delay-line architecture at room temperature**, achieved through a manufacturable SAW device stack combining high mobility of graphene with record high coupling of PMN-PT (demonstration of SAW on PMN-PT is rare in the literature). Here, $\mathcal{R}_{AE}$ is introduced in the revised manuscript as a meaningful figure of merit for AE platform comparison and is defined as AE current normalized to input power and frequency, $I_{AE}/(P_{in} \cdot f)$, with units of $A/(W{\cdot}Hz)$, equivalently C/W. This metric therefore provides a physically intuitive interpretation as the charge generated

per unit input power, enabling more meaningful comparison across AE platforms. The increased AE output is directly relevant to practical acoustic charge transport, RF signal-processing, and sensing applications.

- **Introducing novel backside gate tuning,** leveraging the gate-tunable sheet conductivity of graphene (key 2D-material capability) and high permittivity of PMN-PT which enables output tunability without changing the device geometry. This provides a practical knob for post-fabrication trimming and calibration which is central to platform/device readiness.

Finally, to address the reviewer's concern that the paper is "incremental" from a physics-discovery standpoint: we agree that the underlying AE physics is established; however, *Nature Communications* explicitly notes that applied studies can be evaluated on different criteria than novel-mechanism papers, including technology maturity, benchmarking, and operational readiness of known 2D technologies [1]-[2]. In the revision, we added (1) quantitative benchmarking through the introduction of AE responsivity ($\mathcal{R}_{\mathrm{AE}}$), defined as AE current normalized to input power and frequency, as a figure of merit for cross-platform comparison; (2) clarified the steady-state measurement protocol required for reliable gate-dependent comparison; and (3) included multi-device data to support reproducibility. We believe these additions better position the work as an engineering optimization that improves practical readiness of graphene-based SAW/AE components rather than a new-physics discovery.

Reviewer 2&3

1. In principle, one could always obtain larger AE currents simply by increasing the incident RF power. Therefore, comparing the absolute AE current alone is not physically meaningful. For practical acoustoelectronic device development, the key metric is the AE generation efficiency, i.e., the AE current per unit input power (equivalent to the slope of Fig. 4). I suggest benchmarking this efficiency against previous studies.

Answer to the reviewer:

We sincerely thank the reviewer for this thoughtful and constructive comment. We fully agree that comparing the absolute AE current alone (without accounting for the applied RF drive conditions) can be misleading, and we appreciate the reviewer's suggestion to adopt an efficiency-based metric that is more informative for practical acoustoelectric device development. As established in prior SAW acoustoelectric studies (e.g., [3]), the AE current in the linear (small-signal) regime scales proportionally with the input RF power (SAW intensity) and frequency (dependence set by the SAW wavelength). Motivated by this established scaling, we therefore benchmark performance using AE responsivity ($\mathcal{R}_{\mathrm{AE}}$), introduced and defined here and added to the revised manuscript, as $\mathcal{R}_{\mathrm{AE}} = I_{\mathrm{AE}}/(P_{\mathrm{in}} \cdot f)$, where I_{AE} is the AE current, P_{in} is the input RF power, and f is the operating frequency. This metric has units of $\mathrm{A/(W{\cdot}Hz)}$, equivalently $\mathrm{C/W}$, and provides a more meaningful figure of merit for fair comparison across different AE platforms. Importantly, introducing this figure of merit does not alter our main conclusion: the graphene-on-PMN-PT SAW platform remains superior to previously reported AE platforms and continues to set a new benchmark for AE current generation.

It is also important to note that absolute AE current remains practically relevant, because the maximum deliverable AE output is often constrained by (1) intrinsic saturation/nonlinear effects at high SAW intensity (e.g., due to limited carrier density) and (2) extrinsic power-handling limits of the transducers and RF feed structures. In our case, while the AE response remains linear up to +20 dBm (Fig. 4 in the manuscript), attempts to increase the input power further were limited by device power handling: while some devices survived at 21 dBm, at 22 dBm the IDT RF feed lines were damaged (burnt). Therefore, to provide a complete comparison, we now present both the absolute AE current and the AE responsivity ($\mathcal{R}_{\mathrm{AE}}$) in the benchmarking figure shown below and included in the revised manuscript as Fig. 6.

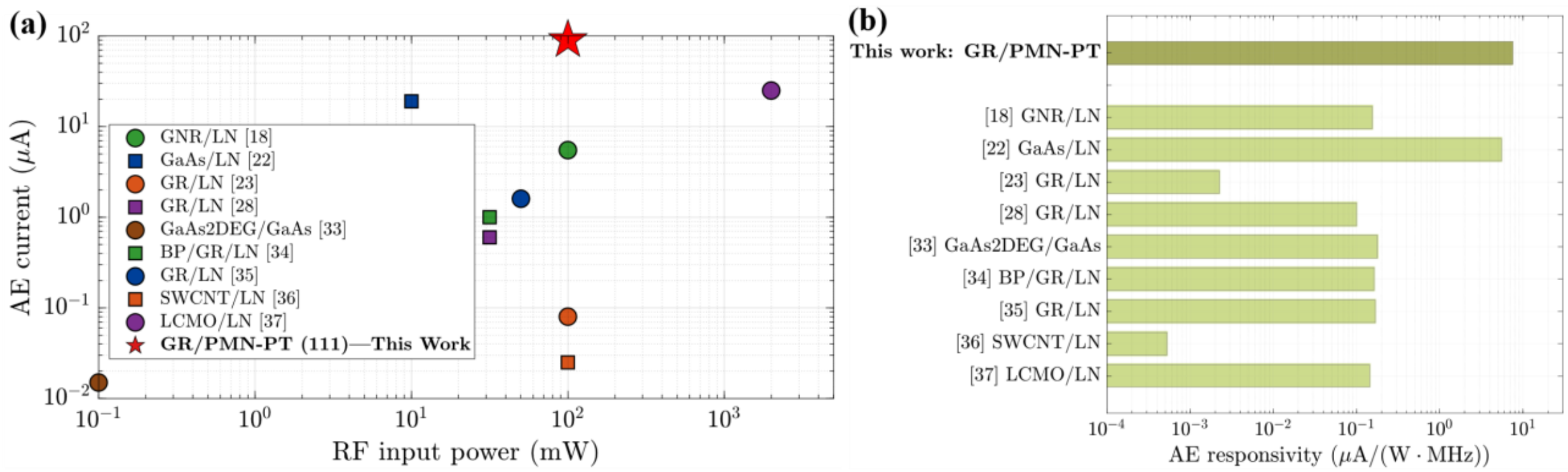

Fig. 1. **Benchmark comparison of acoustoelectric current generation and AE responsivity.** (a) Survey of the highest AE current versus maximum RF power reported across SAW platforms (red star = this work). (b) Comparison of AE responsivity, $\mathcal{R}_{\mathrm{AE}} = I_{\mathrm{AE}}/(P_{\mathrm{in}} \cdot f)$, for the same platforms. $\mathcal{R}_{\mathrm{AE}}$ is introduced here as a figure of merit for cross-platform benchmarking.

2. Another factor limiting AE current/voltage is the saturation of the AE effect at high SAW intensities due to limited carrier density and potential nonlinear effects (see APL 73, 2128 (1998); PRL 82, 2171 (1999); Nano Lett. 25, 14082 (2025)). Have the authors observed any nonlinear behavior in their experiments? If not, they should discuss why the AE current/voltage remains linearly dependent on input RF power even up to +20 dBm.

Answer to the reviewer:

We thank the reviewer for raising the possibility of AE saturation and nonlinear transport at high SAW intensity. No significant nonlinear behavior was observed in our experiments: both the AE current and AE voltage scale linearly with input RF power up to +20 dBm (Fig. 4 in the manuscript), within the measurement resolution. We attribute this linear dependence to operating in the small-signal AE regime, i.e., below the intense-SAW conditions where carrier-density-limited saturation and nonlinear carrier dynamics become significant. Prior studies [4]-[5] show that nonlinear/saturating AE transport emerges when the SAW intensity is sufficiently strong to substantially modulate the carrier distribution; furthermore, the saturation threshold shifts to higher SAW intensity as the carrier density increases.

In our devices, the Hall-measured sheet carrier density is $n_s = 1.31 \times 10^{13}$ cm^{-2}, and the estimated maximum SAW intensity at +20 dBm is ~ 1.63 W/m, which guarantees operation in the linear regime discussed in the cited literature. Notably, the nonlinear regimes reported in [4] were reached due to substantially lower carrier densities ($n < 4 \times 10^{11} cm^{-2}$) and higher SAW intensities ($I > 2.25\ W/m$). Although PMN-PT provides higher electromechanical coupling than LN, the achieved SAW intensity at a given P_{in} still depends on IDT transmission/matching (insertion loss); thus K^2 can lower the equivalent P_{in} for a target intensity with optimized IDTs, but this does not by itself imply nonlinear operation. We also attempted to increase the RF input power beyond +20 dBm to determine the onset of nonlinear behavior, but the devices did not survive, and the IDT RF feed lines were damaged, indicating that our current measurements are limited by IDT power handling rather than intrinsic AE saturation.

3. The authors state K2=0.48 and v_SAW=1962 m/s for (111)-cut PMN-PT based on characterization of SAW delay lines. Please clarify whether these values are experimentally measured or taken from prior literature. Since the electromechanical coupling coefficient is the key parameter responsible for the giant AE effect, I strongly recommend including both experimental and calculation details—possibly in the Supplementary Information—to ensure scientific rigor.

Answer to the reviewer:

We sincerely thank the reviewer for emphasizing the importance of clearly establishing the electromechanical coupling, which is indeed a key factor in the AE response. The values $K^2 = 0.48$ and $v_{SAW} = 1962$ m/s used in our analysis are experimentally extracted from our (111)-cut PMN-PT SAW delay lines (now explicitly stated in the paper), and not from literature. Specifically, v_{SAW} is obtained from time-of-flight (ToF) measurements of nominally

identical delay lines with two different acoustic path lengths, yielding the electrically free-surface velocity v_{free}. The coupling K^2 is then extracted using the standard velocity-based method by comparing v_{free} to the velocity under a short-circuited (metallized) surface, implemented by adding a continuous metallization in the acoustic gap; this yields v_{metal} and therefore $K^2 = ({v_{free}}^2 - {v_{metal}}^2)/v_{free}^2$.

To ensure scientific rigor and transparency, we have added a dedicated section in the Supplementary Information detailing the device variants, ToF extraction procedure, and the full expressions used (Supplementary Eqs. (1)–(4)), along with the measurement setup (Supplementary Fig. 2) and the extracted ToF/coupling values (Supplementary Table 1).

4. The manuscript only reports resistance values of 1.3-2.3 kΩ for the graphene sample, without showing any transfer curve. I suggest including the transfer characteristics and other basic electrical measurements to allow more intuitive comparison with the AE performance.

Answer to the reviewer:

We thank the reviewer for this helpful suggestion. We agree that reporting only a resistance range (1.3-2.3 kΩ) does not fully convey the electrical characteristics relevant to the gate-tunable AE response. In the revised manuscript, we therefore have added a section on graphene characterization and included the graphene transfer curve measured as resistance versus back-gate voltage, together with the corresponding AE current and AE voltage (measured at $P_{in} = 10\ dBm$). Importantly, the measured AE responses are compared against the analytical model using the experimentally measured resistance at each V_{bg} as the input to the fit, and we find good agreement between experiment and theory across devices. These results for six geometrically different devices (IDT pitches of 10 and 15 µm, and delay lengths of 200, 300, and 400 µm) are provided in the Supplementary Fig. 1, enabling intuitive comparison between the gate-dependent transport behavior and AE performance. We also clarify that over gate voltages of 0-60 V the resistance increases monotonically and no sign change is observed in AE signal, indicating operation remains below the Dirac point ($V_D \gg 60\ V$) in our tested range.

While for showing the expected conductivity-tuning trend we have limited V_{bg} to 60 V to avoid applying a high electric field opposite to the poling direction, which could risk depoling or device damage, for one representative device, we extended the sweep to 80 V and the resistance continued to increase, which further corroborates that the Dirac point occurs above our standard sweep range.

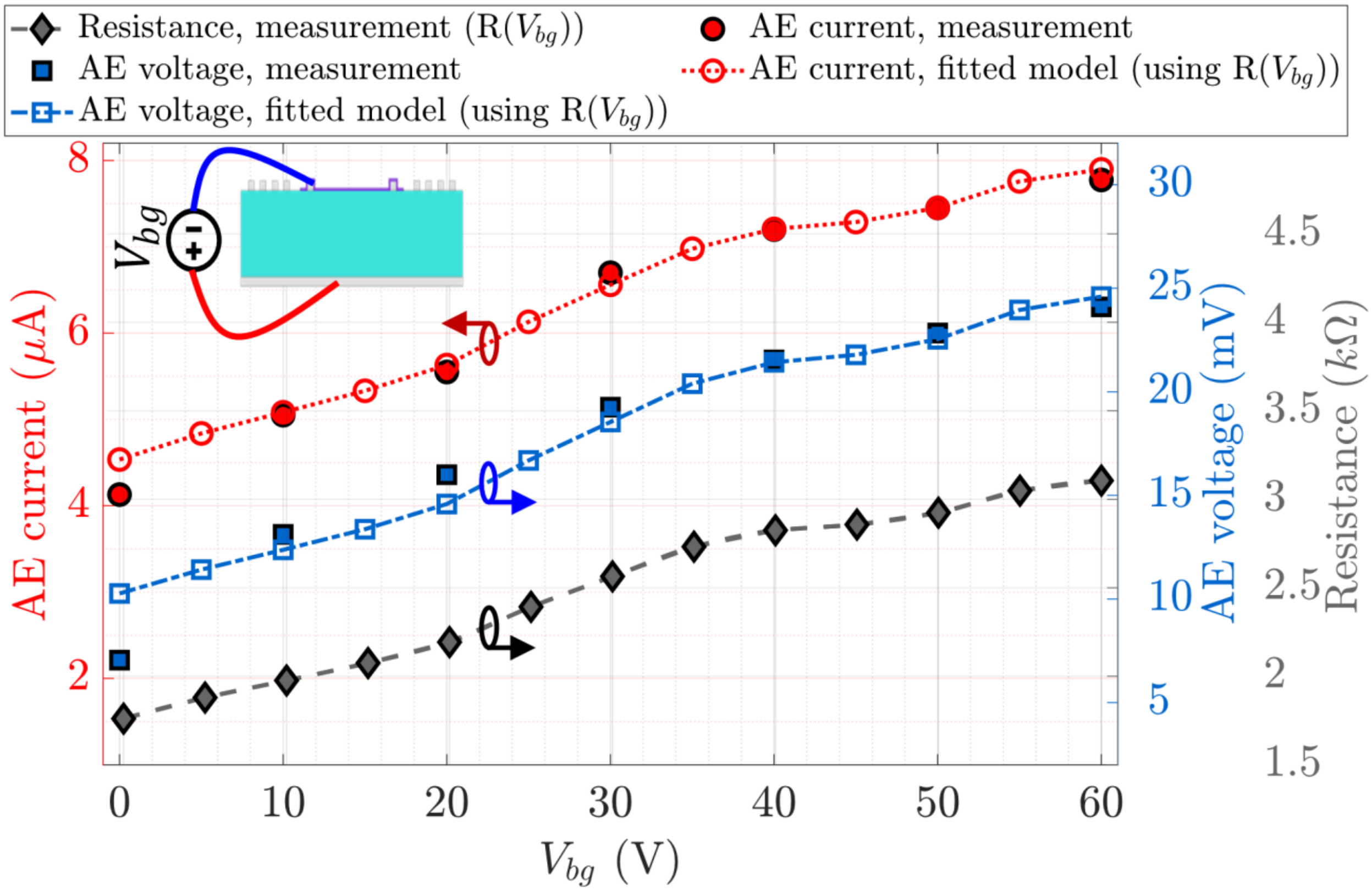

Fig. 2. **Gate-dependent graphene transport and AE response (representative device, P_{in} = 10 dBm).** Measured graphene resistance $R(V_{bg})$ (gray diamonds) compared with the corresponding acoustoelectric current I_{AE} (solid red circles) and acoustoelectric voltage V_{AE} (solid blue squares) as a function of back-gate voltage V_{bg}. Hollow circles/squares show the analytical model predictions for I_{AE} and V_{AE} using the experimentally measured $R(V_{bg})$ at each gate bias as the model input, demonstrating good agreement between experiment and theory. Dashed curves are for visual guidance.

5. At the end of Section II, the statement "It is worth noting that charge carrier mobility has been shown to remain independent of back-gate voltage [28]" is inaccurate, as Ref. 28 shows **clear deviations near the charge neutrality point.** In Section IV, the mobility value is directly adopted from the reference. I recommend including experimental mobility extraction for the actual samples to support the analysis. Does the measured mobility quantitatively fit Equation 3, given that the attenuation and sound intensity are experimentally accessible?

Answer to the reviewer:

Thank you for pointing this out. Ref. 28 in the manuscript [6] shows noticeable deviations in mobility in the vicinity of the charge neutrality (Dirac) point, so the previous statement was incorrect. As mentioned earlier, our devices are operated in a gate-voltage range (0-60 V) that is sufficiently far from the Dirac point; therefore, it is reasonable to assume an approximately constant mobility over the operating regime considered here (i.e., away from the charge-neutrality region where strong carrier-density-dependent effects are expected).

Regarding experimental validation, we agree that extracting mobility for the actual samples would strengthen the discussion and the quantitative use of Eq. (3) in the manuscript. Hence, we have now measured mobility using a Hall/Van der Pauw setup and obtained a mobility of ~400.40 cm²/(V·s), which we use as a representative value for the operating regime. Unfortunately, accessible setups do not support simultaneous back-gating during measurement, however as established earlier, we are operating far from Dirac point, validating the assumption of constant mobility.

Finally, with respect to quantitative comparison using Eq. (3) (in the manuscript), we first estimate the SAW intensity from the applied RF input power and the measured insertion loss (approximating the acoustic power as the

input power reduced by half of the insertion loss [7]). Using this estimated SAW intensity, we fit the model parameters in Eq. (3), and the resulting model captures the measured AE response well. We have revised the manuscript to clarify this approach; reemphasizing that the constant-mobility assumption is applied specifically because the device is operated away from the Dirac point.

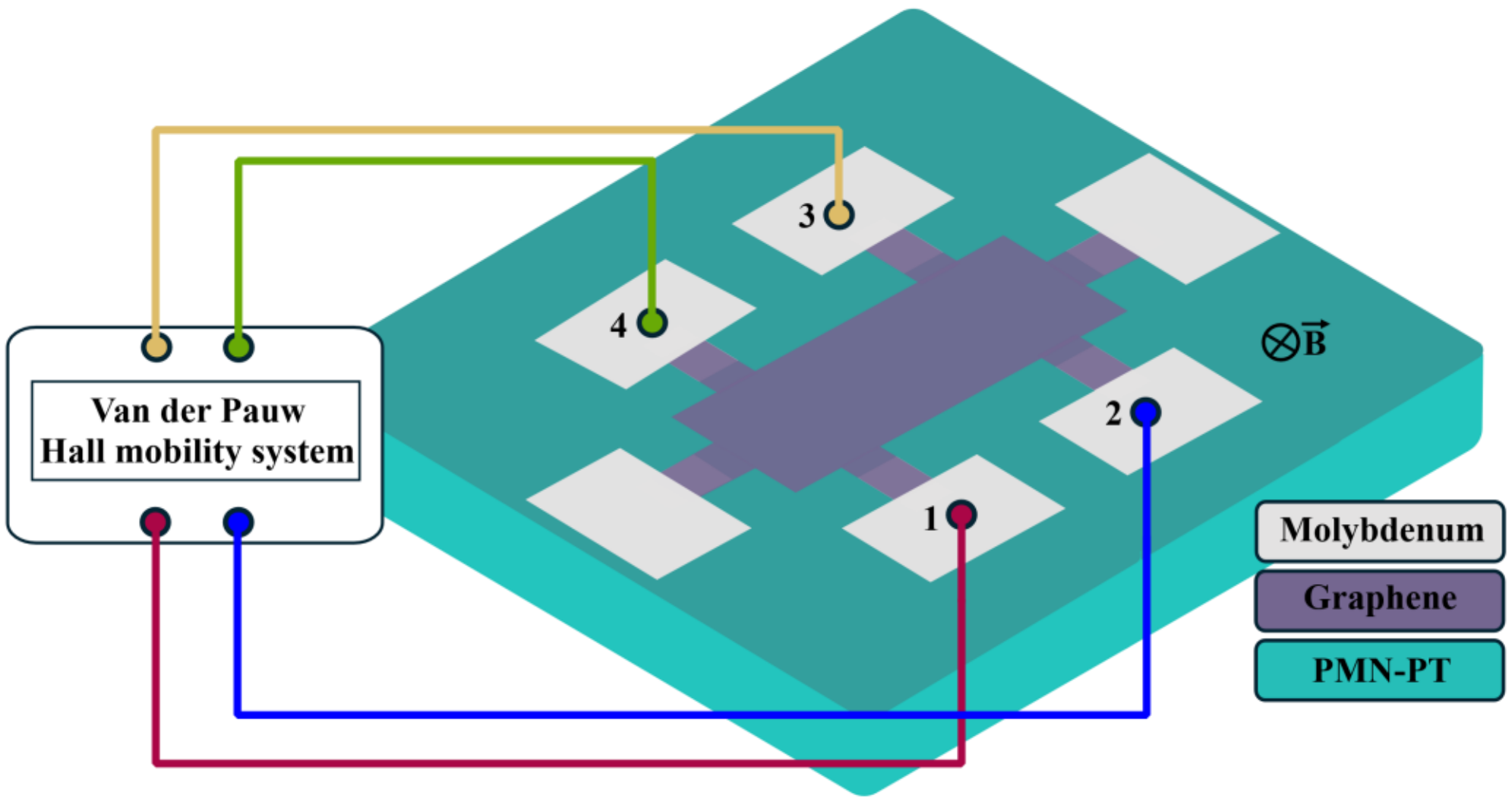

Fig. 3. **Van der Pauw Hall mobility measurement setup.** Schematic of the Hall characterization configuration used to extract the graphene sheet carrier density and mobility using a van der Pauw Hall system. Four contacts (1-4) are connected to the measurement unit while an out-of-plane magnetic field B is applied. The measured stack consists of patterned graphene on molybdenum contacts on a (111)-cut PMN-PT substrate.

Table I. Hall/Van der Pauw electrical characterization of graphene. Extracted sheet resistivity, Hall coefficient, sheet carrier density, and Hall mobility for the measured graphene sample at room temperature.

Sheet resistivity ($\Omega/\square$)	Hall coefficient (cm^3/C)	Sheet carrier density (cm^{-2})	Mobility $cm^2/(V \cdot s)$
1188.84	0.0476	1.31×10^{13}	400.4

6. How is the theoretical curve in Fig. 5 obtained? Specifically, how were the capacitance Cg and mobility μ values in Equation 4 determined? More details should be provided.

Answer to the reviewer:

We thank the reviewer for requesting clarification on how the theoretical curves in Fig. 5 of the manuscript are obtained. To clarify the role of Eq. (4) in the manuscript, it provides the expected electrostatic link between back-gate voltage and carrier density, $n(V_{bg})$, and therefore the qualitative trend of graphene conductivity with gating. The gate capacitance per unit area is $C_g = (\varepsilon_0 \varepsilon_{zz}^T)/t$, where $\varepsilon_{zz}^T = 640$ (Ref. 19 in the manuscript [8]) and the PMN-PT thickness is $t = 500\ \mu m$ giving $C_g = 1.13\ nF/cm^2$. We also report our room-temperature Hall mobility, $\mu = 400.4\ cm^2/(V \cdot s)$ (Supplementary Table 2). For the theoretical curve in Fig. 5 of the manuscript, however, we do not calculate $\sigma(V_{bg})$ from C_g, V_{Dirac}, or μ; instead, the measured steady-state $R(V_{bg})$ is converted to $\sigma_{\square}(V_{bg})$ and directly fed to the model. Accordingly, parameters such as C_g, V_{Dirac}, and μ are provided for physical context and intuitive understanding, rather than as input fitting parameters.

We would also like to mention that during the revision, we identified that gate-dependent measurements are affected by a settling time which has caused an artifact presented in the first draft of the manuscript. We have now corrected our measurement in the revised manuscript and further discussed that matter in supplementary notes. Initially, the observed gate-dependent changes in AE current/voltage which increased with gate voltage and peaked at ~50 V followed by a decline was incorrectly attributed to the possibly of reaching the Dirac point. After repeating the

experiments more extensively at various recording durations, we found that following each V_{bg} step the graphene resistance $R(V_{\text{bg}})$ evolves with a large settling time constant (potentially due to the charge trapping/de-trapping in graphene/PMN-PT interface which is also observed in [10]), and AE current/voltage follow the same slow relaxation. Therefore, measurements taken after short waiting times (~1 min) can underestimate the resistance modulation.

In the revised protocol, for each V_{bg} point we wait until $R(V_{\text{bg}})$ fully stabilizes (~30 min for each point), then measure R, I_{AE}, and V_{AE} at steady state. The theoretical curves are then generated by using the measured $R(V_{\text{bg}})$ (converted to $\sigma_{\square}(V_{\text{bg}})$) as the model input in Eqs. (2)-(3), yielding predicted $I_{\text{AE}}(V_{\text{bg}})$ and $V_{\text{AE}}(V_{\text{bg}})$. With this corrected steady-state dataset, the measured trend (larger AE response at higher R) is consistently in agreement with the model and the trends repeat across six geometrically different devices (Supplementary Fig. 1). We have added the measurement protocol and fitting details to the revised manuscript and Supplementary Information. We note that the best performing device yielding the peak result (90.39 µA) was damaged during repeated testing at high power (20 dBm). However, repeated measurements across six devices ($P_{in} = 10$ dBm) show that after each V_{bg} step a long settling time is required, and the steady-state AE response consistently stabilizes at a higher value than short-wait measurements. Therefore, we believe that the previously reported peak value is actually conservative, and the best performing device could have reached an even higher AE current and voltage under fully settled conditions. The effect of settling time on R, I_{AE}, and V_{AE} is shown in Fig 4, highlighting the consistently lower output values for short wait times versus fully settled cases (more than double for the latter).

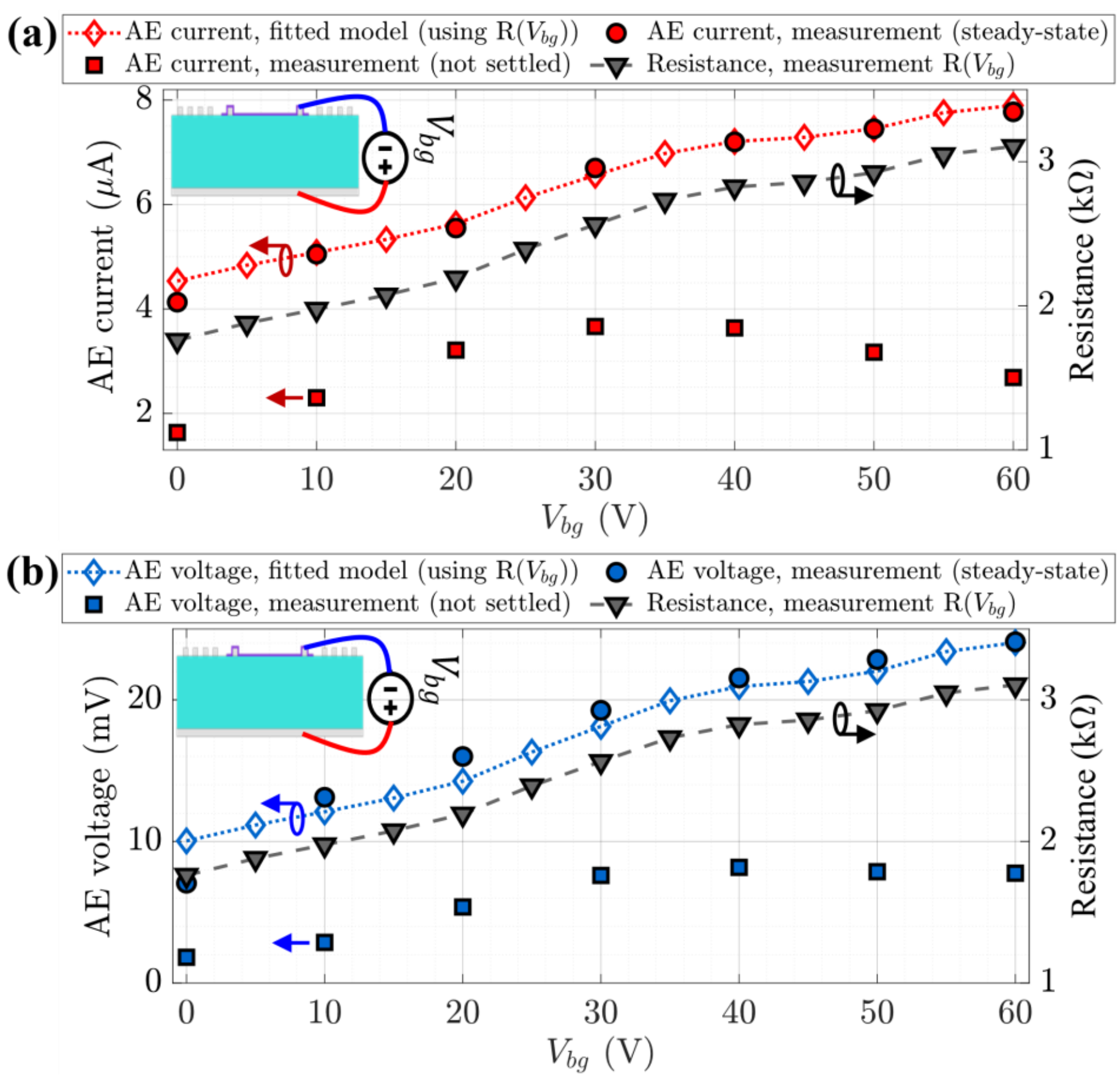

Fig. 4. **Gate-tunable graphene resistance and acoustoelectric response (measured at $P_{in} = 10\ dBm$).** (a) Acoustoelectric current I_{AE} versus back-gate voltage V_{bg} measured in a short-circuit configuration, shown together with the measured graphene resistance $R(V_{bg})$. Solid circle markers denote steady-state values (after sufficient settling time), while square markers show non-settled measurements acquired with a short wait time. The fitted model curve is calculated using the experimentally measured $R(V_{bg})$ at each bias point. (b) Corresponding acoustoelectric voltage V_{AE}

versus V_{bg} measured in an open-circuit configuration, with $R(V_{bg})$ overlaid on the same plot. The monotonic increase in R over 0-60 V indicates operation remains below the charge-neutrality point within the tested range.

7, Some numerical values in the data appear overly precise or coincidental—for example, the reported 50 µA and 90 µA in the captions of Fig. 4 and Fig. 5. What is the measurement resolution? Were the values exactly 50 and 90 µA? Please clarify.

Answer to the reviewer:

We thank the reviewer for pointing this out and agree that the originally stated values could appear overly exact. These numbers were rounded for readability in the initial captions and were not intended to imply exact measured values. In our measurement setup, the resolution is ~10 nA for I_{AE} and ~0.05 mV for V_{AE}. In the revised manuscript, we have updated the captions/text to report the measured values with appropriate significant figures. Specifically, without gate bias the reported AE current/voltage are 61.17 µA / 225.43 mV at 20 dBm without gate voltage and 90.39 µA / 341.72 mV with $V_{bg} = 50$V.

8. There seems to be a typo in the statement "Maximum AE interaction occurs when the two conductivities match (i.e., σ□=σ?)".

Answer to the reviewer:

We thank the reviewer for pointing this out. The intended statement is that "Maximum AE interaction occurs under the conductivity-matching condition, (i.e., $\sigma_{\square} = \sigma$⍰)."

We have corrected the typo in the revised manuscript to ensure the symbol σ_m is displayed consistently.

with λ as the SAW wavelength, $\sigma_{\square}$ as the sheet conductivity of the resistive film, and $\sigma_m = v_{SAW}\left(\sqrt{\varepsilon_{xx}^T\,\varepsilon_{zz}^T} + 1\right)\varepsilon_0$ as a characteristic conductivity determined by SAW velocity and the surrounding dielectric environment. Here, ε_{xx}^T and ε_{zz}^T denote the dielectric constants of the piezoelectric at constant stress conditions. Maximum AE interaction occurs when the two conductivities match (i.e., $\sigma_{\square} = \sigma_{\mathrm{m}}$). The short-circuit AE current density and open-circuit AE voltage are then:

Reviewer4

1. Is there enough detail provided in the methods for the work to be reproduced?

- On page 2, Section II, please clarify what the symbol σ represents, is it referring to σ_m (metal conductivity) or something else?

Answer to the reviewer:

We thank the reviewer for this helpful comment. In Section II, the symbol $\sigma_\square$ refers to the film sheet conductivity (conductivity per square) of the graphene layer. The characteristic conductivity of the SAW substrate is denoted by σ_m and is defined by the SAW phase velocity and the effective dielectric constant of the substrate. AE interaction is maximized under the conductivity-matching condition $\sigma_\square = \sigma_m$.

with λ as the SAW wavelength, $\sigma_\square$ as the sheet conductivity of the resistive film, and $\sigma_m = v_{SAW}\left(\sqrt{\varepsilon_{xx}^T\,\varepsilon_{zz}^T} + 1\right)\varepsilon_0$ as a characteristic conductivity determined by SAW velocity and the surrounding dielectric environment. Here, ε_{xx}^T and ε_{zz}^T denote the dielectric constants of the piezoelectric at constant stress conditions. Maximum AE interaction occurs when the two conductivities match (i.e., $\sigma_\square = \sigma_\mathrm{m}$). The short-circuit AE current density and open-circuit AE voltage are then:

- please provide further detail on the time gating criteria of the SAW data.

Answer to the reviewer:

We thank the reviewer for requesting clarification on the time-gating criteria. The gate window was chosen based on the expected SAW time-of-flight using the experimentally extracted acoustic velocity $v_\mathrm{SAW} = 1962\mathrm{m/s}$. Specifically, the start and stop times were set to capture the earliest and latest arrival of SAW energy between the input and output IDTs, accounting for the finite IDT interaction length along the propagation direction (i.e., the closest and farthest electrode pairs contributing to launch and detection). The gate limits were defined as

$$t_\mathrm{start} = \frac{L}{v_\mathrm{SAW}},\, t_\mathrm{stop} = \frac{L + 4n\,FP}{v_\mathrm{SAW}},$$

where L is the IDT-to-IDT gap (acoustic delay length), n is the number of finger pairs, FP is the IDT finger pitch (periodicity), and v_SAW is the SAW phase velocity. This time window isolates the acoustic response and suppresses electromagnetic feedthrough in the measured S_{21}.

- It would be helpful to briefly explain the graphene transfer process shown in Figure 2, so that readers unfamiliar with the method can follow more easily.

Answer to the reviewer:

We appreciate this suggestion and agree that a brief description of the graphene transfer process will help readers follow the fabrication flow. Accordingly, we have added the following description to the Methods section (Graphene transfer process) in the revised manuscript:

"A polymer-free wet transfer technique was employed for graphene transfer. Chemical vapor deposition (CVD)-grown graphene on copper foil was cut to the desired dimensions for the target substrate and placed on the surface of a 0.1 M ammonium persulfate solution for copper etching, with the sample centered using a polyethylene terephthalate (PET) retainer. After 2-3 hours of etching, a free-floating graphene film was obtained within the central opening of the PET retainer. The etching solution was subsequently replaced several times with deionized (DI) water to remove residual contaminants. The target substrate was then positioned directly beneath the graphene film, and the DI water

was slowly drained through the bottom-side outlet to laminate the graphene onto the substrate. Finally, the graphene-transferred substrate was dried under a gentle flow of nitrogen gas to remove residual water."

- It would also be valuable to comment on the polarization direction of PMN-PT relative to Vbg and discuss its potential impact on graphene conductivity.

Answer to the reviewer:

We thank the reviewer for this valuable suggestion. The (111)-cut PMN-PT substrate was poled with an electric field applied from the top surface toward the backside electrode (top-to-bottom). In our measurements, the back-gate voltage (V_{bg}) is applied between the backside electrode and the graphene, which corresponds to an electric field largely **opposite to the original poling direction** for positive V_{bg}. According to our hall mobility measurements, the graphene is p-doped at room temperature; therefore, applying positive V_{bg} reduces hole density and graphene sheet conductivity, while negative V_{bg} increases hole density and conductivity. Operating with an opposing field can, in principle, reduce the effective polarization and degrade the piezoelectric/SAW response if the field is sufficiently large (partial depoling). For this reason, we limited V_{bg} to 60 V during the systematic measurements to minimize depolarization risk while still enabling clear electrostatic tuning of graphene conductivity.

References

[1] Nature Communications. Publishing 2D materials research towards industrialisation. *Nat. Commun.* **13**, 1918 (2022). https://doi.org/10.1038/s41467-022-29527-7.

[2] Nature Communications. The value of applied science. Nat. Commun. 14, 587 (2023). https://doi.org/10.1038/s41467-023-36307-4.

[3] Poole, T. & Nash, G. R. Acoustoelectric current in graphene nanoribbons. *Sci. Rep.* **7**, 1767 (2017). https://doi.org/10.1038/s41598-017-01979-8.

[4] McSorley, T. J., Simha, K. & Corcoran, J. E. **et al.** Dynamic carrier modulation via nonlinear acoustoelectric transport in van der Waals heterostructures. *Nano Lett.* **25**, 14082–14089 (2025). https://doi.org/10.1021/acs.nanolett.5c03282.

[5] Rotter, M., Kalameitsev, A. V., Govorov, A. O., Ruile, W. & Wixforth, A. Charge conveyance and nonlinear acoustoelectric phenomena for intense surface acoustic waves on a semiconductor quantum well. *Phys. Rev. Lett.* **82**, 2171 (1999).

[6] Urban, F., Lupina, G., Grillo, A., Martucciello, N. & Di Bartolomeo, A. Contact resistance and mobility in back-gate graphene transistors. *Nano Express* **1**, 010001 (2020). https://doi.org/10.1088/2632-959X/ab7055.

[7] Bandhu, L., Lawton, L. M. & Nash, G. R. Macroscopic acoustoelectric charge transport in graphene. *Appl. Phys. Lett.* **103**, 133101 (2013). https://doi.org/10.1063/1.4822121.

[8] Sun, E. & Cao, W. Relaxor-based ferroelectric single crystals: Growth, domain engineering, characterization and applications. *Prog. Mater. Sci.* **65**, 124–210 (2014). https://doi.org/10.1016/j.pmatsci.2014.03.006.

[9] Tang, C. C., Chen, Y. F., Ling, D. C., Chi, C. C. & Chen, J. C. Ultra-low acoustoelectric attenuation in graphene. *J. Appl. Phys.* **121**, 124505 (2017). https://doi.org/10.1063/1.4979207.

[10] Jie, W. & Hao, J. Time-dependent transport characteristics of graphene tuned by ferroelectric polarization and interface charge trapping. *Nanoscale* **10**, 328–335 (2018). https://doi.org/10.1039/C7NR06485C.

Thank you for your consideration.

Sincerely,
Hamed Atashbar
(on behalf of all authors)

Reviewer #2 (Remarks to the Author):

We thank the authors for their efforts in addressing the previous round of comments. Additional measurements have been performed, and the manuscript has been revised to provide further clarification of the experimental procedures. The device characterization is now more complete, and the physical discussions have been expanded. Nevertheless, after careful consideration of the revised manuscript and the authors responses, I remain somewhat disappointed with the current version.

Thank you for acknowledging our effort in addressing the previous comments. We also think that the reviewers' thoughtful comments have helped us in improving the quality of our paper significantly and hope to respond to the new comments succinctly.

1. Figure 6 compares the device performance with other studies. After normalizing by input power, the reported results do not demonstrate a significant improvement than that of Ref. 22 (GaAs/LN).

Answer to the reviewer:

Regarding the significance of the improved performance we would like to point out that the only reported result that come close to our presented result is from Ref. [22] (in the manuscript) over which we are reporting 38% improvement in the AE responsivity (the newly defined acoustoelectric figure of merit) and more than 375% improvement in the absolute reported acoustoelectric current. We respectfully disagree with the respected reviewers in that this improvement is not significant given that over the past 28 years following the reported results in Ref 22, there has not been any work that reported a result coming even close to that record to the best of our knowledge. To better represent the significance of our results we have now modified Fig. 6b in the manuscript by plotting the AE responsivity on a linear scale and by adding the numerical values for each platform.

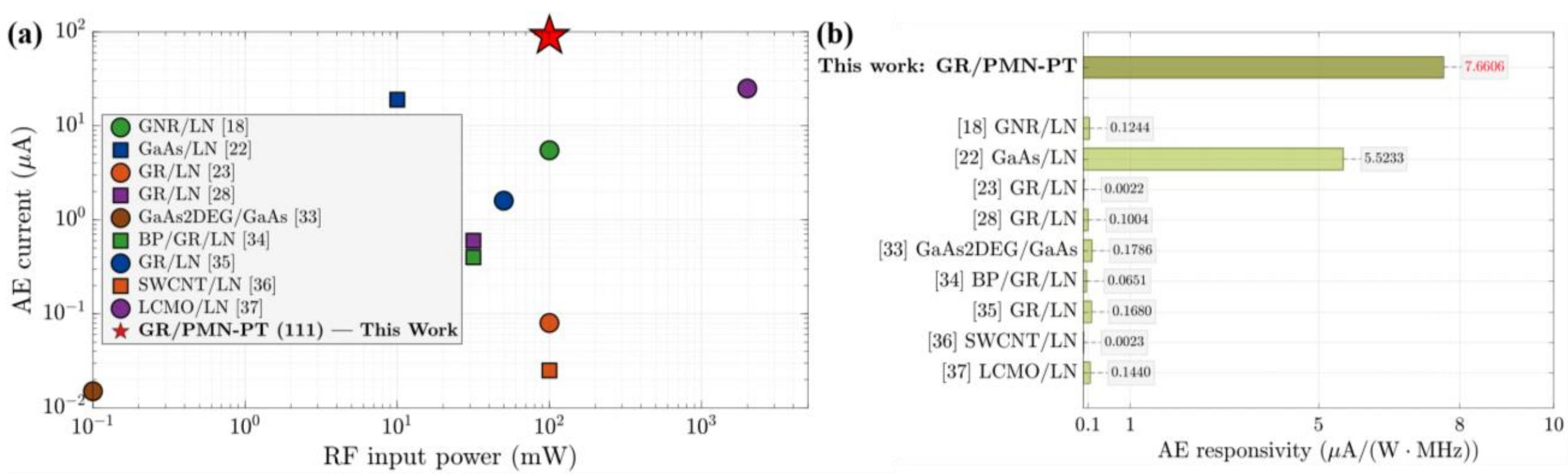

Fig. 1 Benchmark comparison of acoustoelectric current generation and AE responsivity. (a) Survey of the highest AE current versus maximum RF power reported across SAW platforms (red star = this work). (b) Comparison of AE responsivity, $\mathcal{R}_{AE} = I_{AE}/(P_{in} \cdot f)$, for the same platforms. $\mathcal{R}_{AE}$ is introduced here as a figure of merit for cross-platform benchmarking.

2. Compared to other works on the acoustoelectric effect in 2D materials, the apparent enhancement in acoustoelectric response appears to stem largely from the unusually large sample size. In this work, the 2D material fully spans the IDT aperture, whereas most prior studies employ flakes that are typically an order of magnitude smaller than the IDT width. This geometric advantage raises concerns as to whether the observed signal truly reflects intrinsic material properties or is primarily a consequence of the increased interaction area.

Answer to the reviewer:

As stated, it is true that the measured AE current depends on both the material/platform response and the geometry of the 2D electron system interacting with the surface acoustic wave. To address this concern, we added a width-normalized benchmarking analysis in the Supplementary Fig. 4. Before discussing the details of this benchmarking, we would like to point out that one of the great advantages of our technology is the scalability of the platform where as in many other instances the scaling to achieve reasonable and useful outputs stays extremely challenging.

First, we normalized the AE responsivity ($\mathcal{R}_{\mathrm{AE}}$) by the active channel width, using $I_{\mathrm{AE}}/(P_{\mathrm{in}} \cdot f \cdot W_{\mathrm{ch}})$, where W_{ch} is the width of the 2D conducting channel (Fig. 2a). This metric removes the direct contribution of channel width to the measured AE current. Under this normalization, our graphene/PMN-PT platform reaches 0.0295 μA/(W·Hz·m), which is higher than all compared platforms. Compared with Ref. [22] in the manuscript, the second highest value for AE responsivity ($\mathcal{R}_{\mathrm{AE}}$), our width-normalized AE responsivity ($\mathcal{R}_{\mathrm{AE,W}}$) is more than 4.2× larger.

Second, we added a full-aperture projection, defined as $I_{\mathrm{AE}}\ W_{\mathrm{IDT}}/(P_{\mathrm{in}} \cdot f \cdot W_{\mathrm{ch}})$, to estimate the response expected if each 2D material covered the full IDT aperture width (W_{IDT}), shown in Fig. 2b. This comparison gives an optimistic upper-bound estimate for flake-based reports. Even under this projection, our platform remains the highest-performing system.

We also marked Ref. [34] (in the manuscript) separately because it uses a BP/hBN/GR/LN heterostructure based on exfoliated black phosphorus (BP). In that work, the effective BP channel width is about 5 μm, while the IDT aperture is 350 μm. Because exfoliated BP flakes are typically small and irregular in shape (Fig. 3), extending the same BP/hBN/GR stack to the full 350 μm aperture is not straightforward. Therefore, the full-aperture-projected value for Ref. [34] (in the manuscript) should be viewed as an optimistic upper-bound estimate rather than a demonstrated full-aperture device value.

These added comparisons show that the large AE current and responsivity in our work are not solely due to the larger interaction width. Instead, the device benefits from both strong width-normalized AE response and practical full-aperture integration of graphene across the IDT aperture.

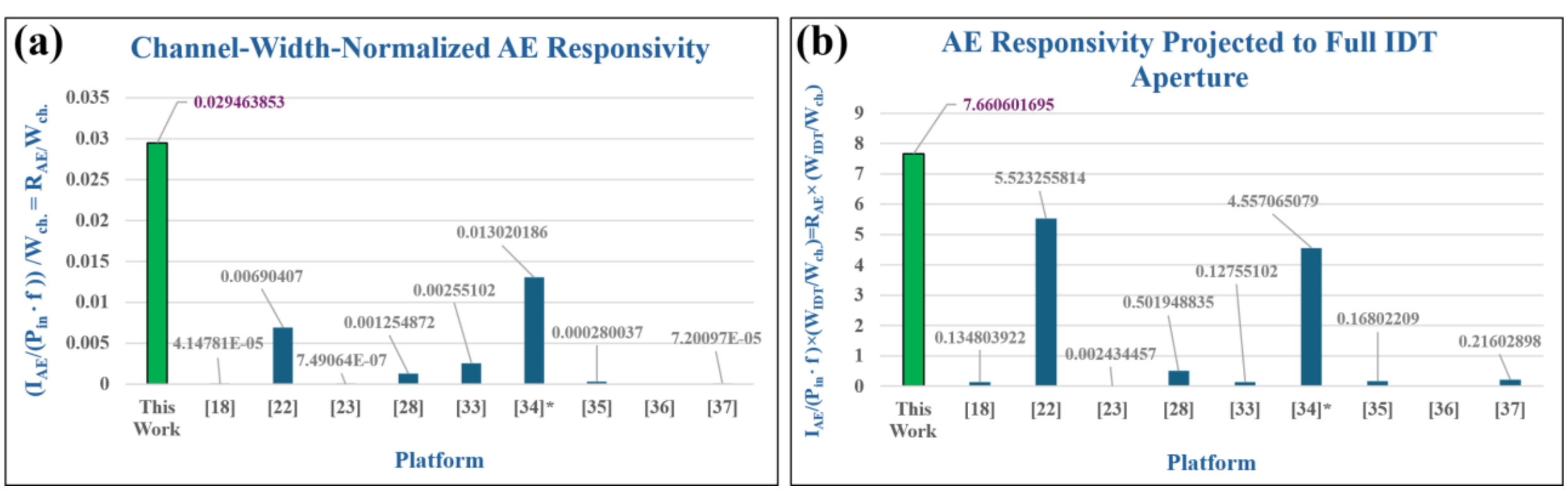

Fig. 2 Width-normalized benchmark comparison of AE responsivity ($\mathcal{R}_{\mathrm{AE}}$). Reference numbers correspond to the numbering used in the main manuscript reference list. **(a)** AE responsivity normalized by the active 2D channel width ($\mathcal{R}_{\mathrm{AE,W}}$), defined as $I_{\mathrm{AE}}/(P_{\mathrm{in}} \cdot f \cdot W_{\mathrm{ch}})$, where I_{AE} is the measured AE current, P_{in} is the RF input power, f is the operating frequency, and W_{ch} is the active channel width. This metric removes the direct scaling of $\mathcal{R}_{\mathrm{AE}}$ with channel width. **(b)** Full-aperture-projected $\mathcal{R}_{\mathrm{AE}}$, calculated as $I_{\mathrm{AE}}W_{\mathrm{IDT}}/(P_{\mathrm{in}} \cdot f \cdot W_{\mathrm{ch}})$, where W_{IDT} is the IDT aperture width. This projection estimates the expected $\mathcal{R}_{\mathrm{AE}}$ if each 2D conducting channel extended across the full IDT aperture. Ref. [34] (in the manuscript) is marked here with an asterisk because the BP/hBN/GR/LN platform used an exfoliated BP channel with an effective width of about 5 μm within a 350 μm IDT aperture; therefore, its full-aperture-projected value should be interpreted as an optimistic upper-bound estimate.

[*REDACTED*]

Fig. 3 Conceptual schematic, false-colored SEM image, and optical micrographs of the BP/hBN/GR/LN acoustoelectric device reported in Ref. [34]. Top left: device schematic. Top right: false-colored SEM image of the BP/hBN/graphene heterostructure. Bottom: optical micrographs showing the FET positioned between the IDTs. Images are adapted from Ref. [34] in the manuscript.

3. In Fig. 3, the authors present a Hall bar device but extract mobility using only the four central contacts via the van der Pauwmethod. This approach is unconventional and potentially problematic: it neglects the influence of the source/drain metal contacts (not used in van der Pauw method) and disregards the complex current distribution inherent to complex Hall bar geometries. These oversights can significantly increase measurement uncertainty and introduce systematic errors. Why was a standard six-terminal Hall measurement not employed instead? Or they can just use the van der Pauw method on a standard square device instead on the Hall bar device.

Answer to the reviewer:

While we agree that a standard six-terminal Hall bar measurement is preferable in principle, practical constraints prevented its execution. However, we argue that this configuration approximates a van der Pauw Greek-cross-like geometry, satisfying van der Pauw’s key conditions to a reasonable degree [1], though we acknowledge it is not a dedicated square or cloverleaf van der Pauw structure. As shown in Supplementary Fig. 3, contacts 1-4 are defined on narrow molybdenum leads overlapping the graphene only over a small area at the tips of the Hall bar arms. This geometry is functionally analogous to a Greek-cross van der Pauw structure, in which narrow arms isolate the measurement contacts from the central active region, a configuration explicitly identified as one of the most accurate van der Pauw geometries for minimizing finite-contact-size errors [2].

In this configuration, the small effective contact footprint satisfies the requirement for sufficiently point-like contacts at the periphery of the active graphene region, rather than metallic shorts within the central region. The source and drain contacts were not used as current or voltage terminals and were electrically floating during the measurement, which minimizes, but does not completely eliminate their possible influence on the local current distribution. The CVD graphene film is continuous and contains no isolated holes; therefore, the measurement satisfies the main van der Pauw requirements to a reasonable degree [1]-[2].

We further note that the ideal van der Pauw formulation assumes infinitesimal contacts on the sample perimeter, and finite contacts can introduce errors by modifying the current distribution and partially shorting the Hall field [3]. Chwang *et al.* showed that finite contacts for a square sample reduce the measured Hall voltage through two main mechanisms: current redistribution caused by finite current/voltage electrodes and current shunting around the Hall voltage contacts. They also showed that these errors decrease as the effective contact size is reduced; for their analyzed square geometry with a contact-size ratio () of approximately $d/a = 1/8$, where d is the effective contact size and a is the square side length, the resistivity correction factor was 1.0025, and the overall Hall mobility correction was below 10%.
Further numerical study showed that Greek-cross sheet-resistor structures are valid and more accurate van der Pauw test structures when the arm length exceeds the arm width, with sheet-resistance errors below 0.1% for suitable Greek-cross geometries [4]. Our structure follows the same design principle: the central graphene rectangle is not metallized or damaged, and the metal-covered arm tips act as equipotential extensions connected to the boundary of the active region, rather than metallic shorts within the central region. The additional source/drain electrodes were electrically floating and therefore did not inject or collect current during the measurement; however, we acknowledge that they could still weakly modify the boundary conditions. Based on these considerations and the dimensions of our layout, we estimate the geometry-related uncertainty in the extracted hall measurements to be on the order of 5-10%.

We would have ideally fabricated a dedicated Greek-cross, square, or cloverleaf van der Pauw test structure on a separate sample for independent verification. Unfortunately, due to changes in project priorities and funding at our graphene provider over the past year, we no longer have access to graphene from the same supply, which makes fabricating additional test devices currently unfeasible. We appreciate the reviewer's understanding of these circumstances.

4, The sheet conductance in Equation 2 actually corresponds to the conductance at SAW frequency. However, the authors substitute experimentally measured DC resistance directly into this expression. Moreover, they totally neglected the contact resistance.

Answer to the reviewer:

We agree that the conductance entering the relaxation model should represent the sheet conductance of the 2-D conducting channel interacting with the SAW field. However, in the small-signal acoustoelectric relaxation model, this quantity is commonly taken as the low-field DC sheet conductance of the conducting layer at the relevant carrier density. For example, Lee *et al.* [5] explicitly define the conductivity term in the graphene acoustoelectric model as the DC conductivity of graphene. Similarly, Poole and Nash [6] also used room-temperature current-voltage measurements to extract sheet resistance values of graphene nanoribbon arrays before comparing the measured acoustoelectric current with the classical relaxation model.

Regarding contact resistance, we agree that the conductance entering Eq. 2 (in the manuscript) should ideally correspond to the graphene channel sheet conductance. In our measurements, the resistance used in the model was obtained from two-terminal I-V characterization between the graphene electrodes, at each V_{bg} after steady-state stabilization. Therefore, this value represents an effective device resistance and may include contact resistance.

We note that the transferred CVD monolayer graphene on the devices show resistance values of 1.3-2.3 kΩ, reflecting device-to-device variability associated with large-area graphene transfer and patterning. Within this experimental spread, the measured $R(V_{bg})$ still captures the monotonic increase in device resistance with gate voltage, which is the key input needed to describe the observed conductivity-matching trend. Accordingly, the model is used here to capture the gate-dependent AE trend rather than to extract a contact-free intrinsic graphene sheet conductivity.

Importantly, the main performance metrics reported in the manuscript, including I_{AE}, V_{AE}, and $\mathcal{R}_{AE} = I_{AE}/(P_{in} \cdot f)$, are directly measured and do not rely on contact-resistance subtraction. The model-experiment agreement across six devices in Supplementary Fig. 1 supports the conclusion that increasing V_{bg} reduces the effective graphene conductance and improves conductivity matching, leading to enhanced AE current and voltage.

5, The authors report requiring ~30 minutes of relaxation at each gate voltage before stable transport data can be acquired. Such as low response is difficult to reconcile even with known charge trapping mechanisms in 2D heterostructures.

Answer to the reviewer:

We thank the reviewer for raising this important point. The ~30 min stabilization time per gate step was experimentally observed during DC back-gate measurements of the graphene/PMN-PT devices.

After each V_{bg} step, the graphene resistance evolved slowly toward a steady state value; therefore, all gate-dependent transport and AE data were recorded only after resistance stabilization at each bias point. We attribute this relaxation to charge trapping/detrapping and charge redistribution at the graphene/PMN-PT interface. This interpretation is consistent with the time-dependent transport behavior reported for graphene on PMN-PT [7], where the resistance evolves gradually before reaching saturation over long time scales (Fig. 4), and where sub-coercive gate bias produced gradual resistance evolution dominated by interfacial charge trapping/detrapping. Since the gate voltages used here, 0-60 V, are below the reported coercive voltage of PMN-PT in that study (~100 V), full polarization switching is not expected to dominate the relaxation. Thus, the waiting protocol ensures that the reported AE response corresponds to the steady-state graphene resistance rather than transient gate-induced drift.

[*REDACTED*]

Fig. 4 Time-dependent sheet resistance of graphene on PMN-PT under and after applied gate bias, showing slow relaxation toward a saturated steady state. Adapted from Ref. [7].

6, Did the authors monitor gate leakage currents? Could Joule heating due to gate leakage contribute to the observed drift or instability? If substantial gate leakage exists, it could also lead to large DC currents flowing through the channel, potentially masking or distorting the true acoustoelectric signal. We suggest the authors: (i) test negative gate biases, and (ii) consider modulating the SAW at low frequency and using lock-in detection to isolate the genuine acoustoelectric response from background drift or thermal artifacts.

Answer to the reviewer:

We thank the reviewer for this important comment. We agree that gate leakage could in principle contribute to background DC current or Joule heating. We therefore checked for leakage-related artifacts.

During PMN-PT poling, a much larger voltage of 750 VDC was applied across the PMN-PT substrate using a Stanford Research Systems PS310 high-voltage power supply. Over the poling period (20-30 minutes), the measured current remained at the supply noise floor, 0-0.001 mA, limited by the instrument's 0.001 mA current resolution. This indicates negligible leakage through the PMN-PT, even at an electric field much larger than that used for back-gate tuning.

For the AE measurements, the applied back-gate voltage was limited to 0-60 V. We also measured the current through the graphene channel under applied back-gate bias without RF excitation/SAW and did not observe a measurable DC current comparable to the AE signal. Therefore, the measured AE current is not attributed to gate leakage or a leakage-driven current through the graphene channel.

Regarding negative gate bias, our preliminary measurements showed that applying negative V_{bg} reduced both the AE current and AE voltage, while preserving the same signal polarity. This behavior is consistent with the p-doped nature of the graphene film: negative back-gate bias increases hole accumulation, moves the graphene farther from the Dirac point, lowers the channel resistance, and therefore moves the sheet conductivity farther from the conductivity-matching condition needed for stronger AE conversion. We therefore focused the gate-dependent study on positive V_{bg}, where the graphene resistance increased monotonically and the AE response improved.

References

[1] Van der Pauw, Leo J. A method of measuring specific resistivity and Hall effect of discs of arbitrary shape. *Semiconductor Devices: Pioneering Papers*. 174-182 (1991). https://doi.org/10.1142/9789814503464_0017.

[2] Lake Shore Cryotronics. *7500/9500 Series Hall System User's Manual: Appendix A, Hall Effect Measurements*. Lake Shore Cryotronics, Westerville, OH.

[3] Chwang, R., Smith, B. J. & Crowell, C. R. Contact size effects on the van der Pauw method for resistivity and Hall coefficient measurement. *Solid-State Electron.* **17**, 1217–1227 (1974). https://doi.org/10.1016/0038-1101(74)90001-X.

[4] David, J. M. & Buehler, M. G. A numerical analysis of various cross sheet resistor test structures. *Solid-State Electron.* **20**, 539–543 (1977). https://doi.org/10.1016/S0038-1101(77)81011-3.

[5] Lee, C. P. et al. Employing graphene acoustoelectric switch by dual surface acoustic wave transducers. *Sci. Rep.* **9**, 8235 (2019). https://doi.org/10.1038/s41598-019-44689-z.

[6] Poole, T. & Nash, G. R. Acoustoelectric current in graphene nanoribbons. *Sci. Rep.* **7**, 1767 (2017). https://doi.org/10.1038/s41598-017-01979-8.

[7] Jie, W. & Hao, J. Time-dependent transport characteristics of graphene tuned by ferroelectric polarization and interface charge trapping. *Nanoscale* **10**, 328–335 (2018). https://doi.org/10.1039/C7NR06485C.

Reviewer #2 (Remarks to the Author):

I thank the authors for their responses to previous comments. I find the clarification regarding the AE response and the width-normalized benchmarking analysis to be satisfactory. Regarding the use of DC resistance in the model, while I understand that this is a common practice in the literature, I suggest the authors explicitly state in the main text that the rigorous approach should utilize the AC resistance at the SAW frequency, and that the use of DC values serves as a valid approximation. Additionally, I recommend briefly discussing the rationale for the ~30-min relaxation time observed in graphene on PMN-PT.

Overall, the manuscript has improved substantially in both completeness and reliability, and I recommend it for publication.

Answer to the reviewer:

We sincerely thank the reviewer for the constructive comments and helpful suggestions, which have improved the clarity and completeness of the manuscript. We also thank the reviewer for considering our work suitable for publication.

The requested discussions have been added to the main text as follows:

1. "After each gate-voltage step, the graphene resistance and the corresponding AE response exhibited a slow relaxation toward steady state; therefore, each bias point was allowed to stabilize before recording the data, typically for about 30 min. This relaxation is likely associated with slow charge trapping/detrapping and interface charge redistribution at the graphene/PMN–PT interface, which can gradually modify the graphene sheet conductivity (Supplementary Note 1)."
2. "Strictly, the acoustoelectric model should use the sheet resistance at the SAW frequency; however, the DC resistance is used here as a valid approximation to capture the gate-dependent conductivity trend, consistent with common practice in the literature."

Thank you for your consideration.

Sincerely,
Hamed Atashbar
(on behalf of all authors)
